# An in-Depth Survey of Visible Light Communication Based Positioning Systems

**DOI:** 10.3390/s16050678

**Published:** 2016-05-12

**Authors:** Trong-Hop Do, Myungsik Yoo

**Affiliations:** School of Electronic Engineering, Soongsil University, Seoul 06978, Korea; dotronghop@gmail.com

**Keywords:** visible light communication, positioning, light-emitting diode, photodiode, image sensor, survey

## Abstract

While visible light communication (VLC) has become the candidate for the wireless technology of the 21st century due to its inherent advantages, VLC based positioning also has a great chance of becoming the standard approach to positioning. Within the last few years, many studies on VLC based positioning have been published, but there are not many survey works in this field. In this paper, an in-depth survey of VLC based positioning systems is provided. More than 100 papers ranging from pioneering papers to the state-of-the-art in the field were collected and classified based on the positioning algorithms, the types of receivers, and the multiplexing techniques. In addition, current issues and research trends in VLC based positioning are discussed.

## 1. Introduction

In the field of positioning, the Global Positioning System (GPS) [1] is the best-known system. When it comes to coverage and cost, no other technologies can compete with it. That being said, GPS is far from a flawless positioning system. Apart from the widely-known disability of GPS in indoor environments, there are cases of GPS failures even in outdoor environments. Although GPS works very well in uncrowded areas, it poses many difficulties for use in metropolitan areas where tall buildings block the lines-of-sight of signal transmissions from satellites. In an experiment conducted in Sydney, only 30% of the test points received signals from a sufficient number of satellites (*i.e.*, three) to calculate the position [2]. WiFi, Bluetooth, radio frequency identification (RFID), and camera-based positioning have been developed to complement GPS. Firstly, these techniques can provide indoor services. Secondly, even in outdoor circumstances these techniques are likely to give a higher accuracy, albeit at higher costs. In recent years, a new kind of technique has emerged, to become a very fruitful area of research on positioning. The technique mentioned here is visible light communication based positioning.

VLC based positioning is a technique that uses visible light signals for determining the location of mobile devices and it has many advantages over RF based positioning. VLC based positioning systems can be installed inexpensively since they utilize existing lighting systems with very few modifications applied. Visible light positioning can be safely used in many places where RF cannot. For example, similar to VLC which can be used in hospital without causing any interference to MRI scanners [3], VLC based positioning can be deployed in hospital. It can also be used in other RF-inappropriate environments such as underwater [4,5] and in underground mines [6]. Another major advantage of VLC based positioning is that compared to RF, the visible light is less subject to multipath effects and thus make the propagation of visible light is more predictable [7,8]. Existing studies reveal that high accuracy can be achieved with VLC based positioning. Beside these advantages, the main motivation for the increasing amount of research on VLC based positioning is the prospect of LED devices and lighting systems becoming ubiquitous, setting the stage for VLC using LEDs in the near future. With many positive features such as cost-effectiveness, long life-time, ruggedness, environmentally friendliness, and greatly controllability, LEDs have been considered to be the lighting device for the 21st century. With the development of LEDs, VLC using LED light has also become the candidate for future wireless technology. It is believed that VLC is just the technique for enabling the Internet of Things (IoT). In the near future, VLC base stations, which also serve as lighting devices, might be installed everywhere, unfold the potential of utilizing them for a pervasive positioning system. Once LED lighting systems are installed everywhere, VLC based positioning can provide a seamless positioning service within much larger coverage than current techniques.

In recent years, VLC based positioning has become an attractive topic and hundreds of papers in this field have been published. However, there are few survey works on VLC based positioning. In [8], the concept of positioning based on VLC and its applications were introduced. However, the paper did not present any positioning algorithms or technical aspects of VLC based positioning. The authors of [9] classified and compared some VLC based positioning techniques. However, each technique was explained in very little detail. Also, the vision based positioning technique was not mentioned in the paper. In [10], four positioning methods using VLC were presented and compared. The limitations of these methods were also pointed out. However, the paper did not mention Time of Arrival (TOA), Time Difference of Arrival (TDOA), and Angle of Arrival (AOA), which are among the most popular algorithms in the filed of VLC based positioning. In [11] an overview of the current status of VLC based indoor positioning systems is provided, with some additional papers being collected. However, very little detail on the positioning mechanisms was presented in that paper. A more detailed survey on VLC based positioning was given in [12]. However, that paper was focused on finding out the potentialities and challenges of using VLC for outdoor positioning so that a numerous papers related to indoor positioning using VLC were not taken into account. Also, some positioning algorithms such as proximity and fingerprinting was not mentioned in the paper. The authors in [13] provided quite an intensive survey on VLC based positioning techniques. However, there was a focus on the comparison between AOA and vision analysis algorithms. Therefore, other algorithms used in VLC based positioning were not surveyed deeply. And finally [14] provided an in-depth survey on VLC based positioning but there are still some algorithms such as proximity and TOA were not covered in the paper.

In this paper, an in-depth survey on VLC based positioning systems is given. More than 100 papers, ranging from the pioneering papers to the current state-of-the-art, were collected and classified based on certain criteria. A detailed comparison among existing techniques is given. Current issues and research trends on VLC based positioning is also discussed.

## 2. Fundamental of Visible Light Communication Based Positioning

### 2.1. Basic Principles and Specific Characteristics

As explained earlier, VLC based positioning is the technique of using the VLC signal transmitted from base stations to find the position of a mobile device. Usually LEDs are used as the VLC transmitters while a PD or an image sensor is used for receiving the positioning signal, which might contain the ID or the geographical location of the LEDs, or any and all information useful for the positioning process.

**Figure 1:**
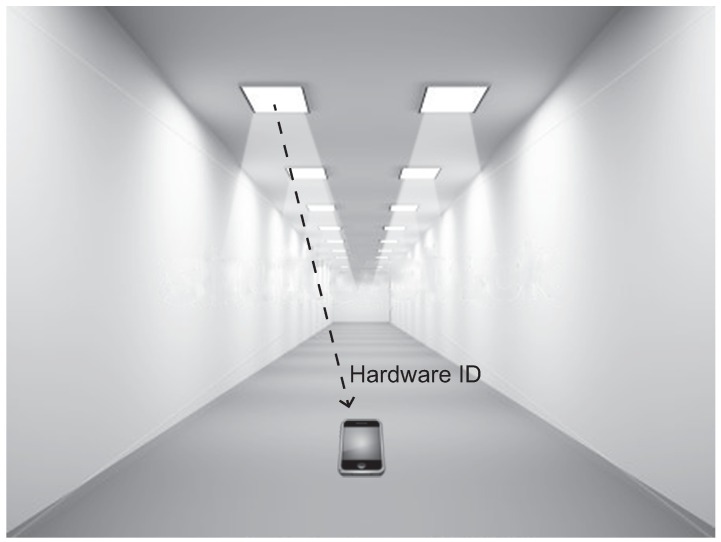
A model of indoor visible light communication (VLC) based positioning.

Figure 1 describes a typical VLC based indoor positioning system where the LED lamps whose original function is to shed light are now used for transmitting a positioning signal, which only contains the ID of LED lamps. Note that the information about the location of the LED lamps is always required for determining the position of the mobile device. In this case, the mobile device might need to obtain such information from its (pre-installed) database which stores the locations of the LED lamps corresponding to their ID.

When a camera is used as the receiver, VLC based positioning might look similar to the vision based positioning [15,16,17,18] which has been developed in robot vision or augmented reality applications, for quite long time. The key difference between the two techniques is the manner in which the mobile device obtains positioning information. While in VLC based positioning using a camera, this information is obtained by receiving VLC signals actively transmitted from the LED base station. With the conventional vision based positioning, this information is obtained by processing images of (natural or artificial) landmarks in the positioning environment. Even if LEDs are used as artificial landmarks, vision based positioning still differs from VLC based positioning in that these LEDs only radiate unmodulated light which does not convey any information.

### 2.2. Terminology

In the literature, the terminologies for VLC based positioning are quite confused. For example, while [9] and [12] used the term “VLC based positioning”, [8] used a shorter and somehow friendlier term “visible light positioning” or “VLP”, [13] even used the confusing term “optical wireless location” or “OWL” to refer to positioning systems based on VLC. Since the uniqueness of all positioning systems mentioned here comes from the use of VLC for determining the location, we suggest that the phrase “VLC based positioning” be used to distinguish the scheme from vision based positioning or infrared positioning.

### 2.3. Taxonomy of VLC Based Positioning Systems

In existing survey works, positioning systems are usually classified based on following criteria:

- The main medium used for determining the location:visible light, infrared, WiFi, Bluetooth, radio frequency, ultra-sound, vision, mechanical energy (inertial or contact), magnetic field, atmospheric pressure, *etc*.
- The type of application: indoor, outdoor, underwater, vehicle, *etc*.
- The algorithm used for determining the location: TOA, TDOA, RSS, AOA, fingerprinting, vision analysis, *etc*.

Since this paper focuses on VLC based techniques, the first criterion mentioned above will not be chosen for the classification. Regarding to the type of application, it is true that VLC based positioning can be classified into techniques designed for indoor, outdoor, underwater, or vehicle environment. However, most existing VLC based positioning techniques aimed to indoor applications. Therefore, the second criterion is also inappropriate for the classification. Instead of that, the VLC based positioning systems in this survey will be classified mainly based on the algorithm used for determining the location. In addition, classifications based on less distinguishable criteria such as the type of receiver and the multiplexing techniques will also be provided.

## 3. Algorithms in VLC Based Positioning

Basically positioning techniques using radio waves, ultra sound or other media can all be adopted for use with VLC. In [19], wireless indoor positioning techniques were classified into three groups: triangulation, scene analysis (also known as fingerprinting), and proximity. Other researchers [20] divided indoor position estimations based on four techniques: triangulation, fingerprinting, proximity, and vision analysis. This paper classifies VLC based positioning techniques into five groups: proximity, fingerprinting, triangulation, vision analysis, and dead reckoning. The proximity and fingerprinting techniques are chosen to be introduced first because of their simplicity in implementation and low accuracy in estimation compared to the others.

### 3.1. Proximity

The simplest location sensing technique is proximity, which cannot give absolute or relative positions but only proximity location information. In this technique, the proximity location of the mobile device is determined based on the signal from a single LED base station. Each LED base station transmits its identification (ID) code which will be received by the mobile device. Each ID is associated with a specific location of LED base station and this association is stored in a database. When receiving the light from a LED base station with a certain ID, the mobile device will look up the location associated with this ID in the database. The position of the mobile device is then determined as the whole area covered by the light radiated from the found LED base station.

Although being simple to implement, proximity has many useful applications. In [8] a system is presented for tracking the positions of assets like wheelchairs or portable medical devices in a hospital. After detecting the ID of the nearest LED base station, the mobile device sends this information, via ZigBee or WiFi, to the central computer on which is maintained a database of the IDs of the LEDs and their positions. Then, the location of the LED at which the mobile device is currently collocated, or even the map of the corresponding hall is send back to the mobile device. A proximity systems using VLID and Zigbee wireless network were tested in [21,22,23]. Firstly, the LED base stations transmitted their VLID to a PD-equipped mobile device. The mobile device transmitted the ID to the center node via a Zigbee wireless network. The center node received the ID and looked for the position corresponding to that ID. Then it transmitted the position information back to the mobile device, also via Zigbee, and thus the mobile device found its proximity.

In [24], the visible light Cell-ID was used for channel allocation. In [25], several realistic VLID based navigation systems used in underground subway stations and in a super market were demonstrated.

The report [26], which is one of the first studies on VLC based positioning, proposed the idea of using an ‘intelligent’ traffic light to transmit signals for vehicle navigation. And [27,28] realized this idea by demonstrating an LED location beacon system with which LED panel traffic lights transmit light patterns to the camera-based receiver on the vehicle. Upon receiving and matching the light patterns, the location codes can be obtained.

A low cost indoor navigation system using LED lamps to transmit IDs to a PD attached on a smart phone was proposed in [29]. The ID then was translated to a location through a correspondence table between ID and location installed on the smart phone beforehand. The data was transmitted using OOK modulation. Manchester coding was employed to prevent flickering. Dimming was achieved through pulse width modulation. Experimental result showed that the position of the smart phone can be determined within a range of 4.5 m.

In [30], an architecture of localization using visible light ID and digital mapping is presented. A PD attached to a smart phone was used to receive the light ID. The paper showed that the proposed technique can be used to navigate the person to a desired location, as well as for indoor tracking.

An indoor navigation systems for visually impaired people was proposed in [31,32]. LEDs installed in the pathway ceiling were used to transmit visible light ID to a portable device worn by the user. After that, the ID was sent to an external server and positional information, for example, latitude and longitude were sent back to the user. The experiment results reported an accuracy of 1–2 m obtained by the system.

Fluorescent light was used instead of LEDs in [33], which also presented an indoor positioning system for visually impaired people. In this system, a photodiode was used to receive the position information transmitted from fluorescent lamps installed on the ceiling. Then a PDA would transmit this information to the user through its speaker.

Usually, proximity only provides an approximate position of the mobile device, that is the area covered by the LED light. Depending on the system, this coverage area can be quite small as in case of the asset tracking system in the hospital in [8], or much larger as in case of the vehicle positioning system in [27,28]. In either case, the distance error might be at least a few meters, too far to be accepted for applications that require good positioning. However, with the help from additional devices the estimation accuracy provided by proximity can be substantially increased.

**Figure 2:**
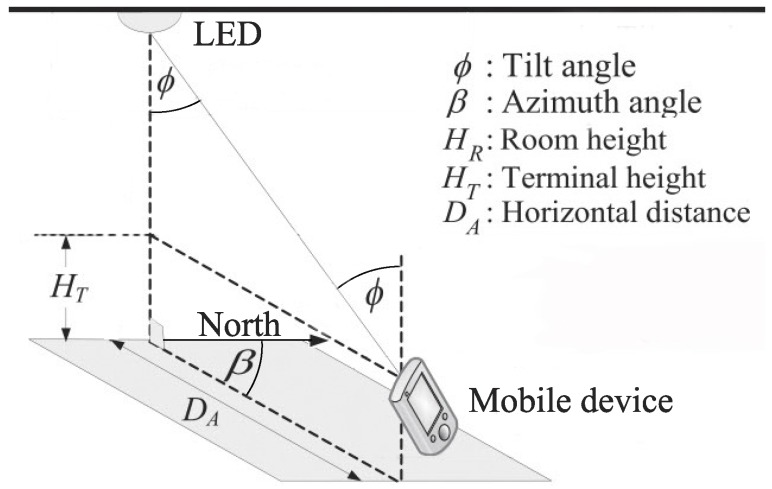
Positioning system using VLID and 6-axis sensor.

**Figure 3:**
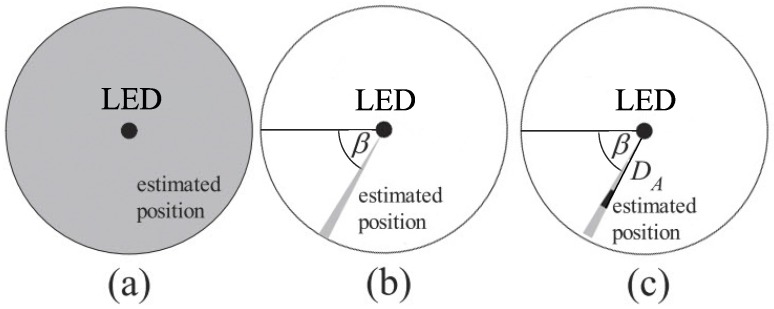
Estimated position by (**a**) VLID; (**b**) VLID and azimuth angle; and (**c**) VLID, azimuth, and tilt angle.

In [34,35,36] a simple, yet effective method was proposed for using a 6-axis sensor to reduce the distance error of a VLID system down to the range of 300 to 600 mm. The system, as illustrated in Figure 2, includes a mobile device equipped with a 6-axis sensor and a PD receiving ID signals from an LED base station installed in the ceiling. The 6-axis sensor is capable of measuring the pose of the mobile device in terms of azimuth and tilt angle. To estimate the position, one should directly point the mobile device toward the LED. Given a deliberately small field of view of the PD, the tilting angle of the mobile device, which is measured by the 6-axis sensor, is assumed to approximate the actual angle from the mobile device to the LED. If the height of mobile device $H_{T}$ is known, the horizontal distance $D_{A}$ between the LED and the mobile device can be determined. Figure 3 shows the improvement of the positioning accuracy when using 6-axis sensor. With only data from VLID, the estimated position will be the entire coverage area of the light source as shown in Figure 3a. When the azimuth angle *β* and the horizontal distance $D_{A}$, which is determined through the known tilt angle *α*, are both known, the estimated position will be reduced to much smaller areas as shown in Figure 3b,c.

An advanced proximity system in which multiple LEDs were used to improve positioning accuracy was proposed in [37]. In this system, the output light was shaped by placing a converging lens in front of the array of LEDs. Then, several regions of overlapping light were created. Each region was distinguished by the presence or absence of light from specific LEDs. A single PD with wide field of view was used as receiver. The light from each LED was modulated into square waves at different frequencies between 4 kHz and 10 kHz for multiplexing. The experimental results showed that accuracy depended on the number of LEDs as well as the distance between the LEDs and the lens. When the number of LEDs increases, the number of uniquely defined regions also increases and so does the positioning accuracy. For 9 LEDs there were 655 regions within an area of 5 m × 5 m and the average error was 12.9 cm. Besides the high accuracy, this system has the advantage that it does not require the receiver to directly point toward the LED as in [34,35,36]. However, this system also has the disadvantage that it might create unwanted contrastive highlight and shadow regions in the floor.

### 3.2. Fingerprinting

Fingerprinting, or scene analysis, consists of positioning techniques estimating the relative position by matching online measured data with pre-measured location-related data. Given the irregularities in the distributions of base stations, the indeterminism in the presence of barriers in the environment, and even the inherent variance of each base station, the features (fingerprints) of measured data vary at different locations. For example, due to the uneven distribution of LEDs, the reflections and scatter of light by the wall and by appliances in the room, and even due to the variance in the transmitted power of each LED, the received power varies at different positions in the room. Fingerprinting relies on these differences to estimate the position.

There are two phases in fingerprinting algorithm: online phase and offline phase. In the offline phase, the location related data (e.g., the received signal strength) at each place in the environment is collected. In the online phase, the currently measured data is matched to the previously collected dataset to estimate the relative position. Pattern recognition techniques such as probabilistic, *k*-nearest-neighbor (*k*NN), and correlation can be used for matching the current data and the prior data set [38].

#### 3.2.1. Probabilistic Methods

Suppose that $L_{1}$, $L_{2}$, $L_{3}$, …, $L_{n}$ are *n* location candidates and *s* is the signal strength vector measured on the online phase. The location estimation now becomes the problem of choosing the most appropriate location $L_{i}$ among *n* candidates based on the posteriori probability. Let $P(s|L_{i})$ denote the probability that the received signal strength vector is *s* given the mobile device locates at $L_{i}$, $P(L_{i})$ denote the probability that the mobile device locates at $L_{i}$ regardless any other information, and P(s) is the probability that the received signal strength vector is *s* regardless any other information. Then the probability that the mobile device locates at $L_{i}$ given the received signal strength vector is *s* can be calculated using Bayes, formula [38]:

(1) $$\begin{array}{c} P\left(L_{i},s\right)=\frac{P\left(s|L_{i}\right)P\left(L_{i}\right)}{P(s)} \end{array}$$

For every location candidate $L_{i}$, the posteriori probability $P(L_{i}|s)$ is calculated. The location $L_{i_{0}}$ is determined as the location of the mobile device if the value of $P(L_{i_{0}}|s)$ is highest. When all location candidates are equidistant (*i.e.*, all location candidates are uniformly distributed in the environment), one can assume that the values of $P(L_{i})$ are the same for all $L_{i}$. Obviously, the value of P(s) is the same for all $L_{i}$. Therefore, in this case the decision rule can be simplified to the comparison between the likelihood of each location candidate.

Usually, the likelihood $P(s|L_{i})$ is calculated based on data collected at this location during the offline phase. Assuming that at each location, *k* samples of received signal strength were measured during the offline phase, then the likelihood of each location candidate has a Gaussian distribution with the mean and standard deviation calculated from the *k* sample value of the received signal strength. To achieve better classification among location candidates, the measured data should not be a scalar but a vector of multiple units. For example, the measured data could be a vector $s=(s_{1},s_{2},s_{3},\ldots ,s_{m})$ of received signal strengths from *m* base stations. In this case, a mechanism for extracting the strength of the signal from each base station is required. Supposing that the base stations are independent from each other, the overall likelihood of each location candidate can be calculated as the product of the likelihood of the received signal strength of all base stations:

(2) $$\begin{array}{c} P\left(s|L_{i}\right)=P\left(s_{1}|L_{i}\right)\times P\left(s_{2}|L_{i}\right)\times \ldots \times P\left(s_{m}|L_{i}\right) \end{array}$$

where $P(s_{j}|L_{i})$ denotes the probability that the received signal strength from the $j^{th}$ base station is $s_{j}$ given that the mobile device locates at $L_{i}$.

In practice, the mobile device can be located at anywhere other than discrete locations $L_{1},L_{2},\ldots ,L_{n}$. The more accurate positioning coordinates can be interpolated as the weighted average of the coordinates of all location candidates:

(3) $$\begin{array}{c} \left(\hat{x},\hat{y}\right)=\sum_{i=1}^{n}\left(P(L_{i}|s\right)\left(x_{L_{i}},y_{L_{i}}\right) \end{array}$$

Similar to the decision rule, if all location candidates are equidistant, Equation (3) can be simplified to:

(4) $$\begin{array}{c} \left(\hat{x},\hat{y}\right)=\sum_{i=1}^{n}\left(P(s|L_{i}\right)\left(x_{L_{i}},y_{L_{i}}\right) \end{array}$$

One of the main challenges to the fingerprinting techniques is that the received signal strength can be affected by unpredictable obstructions. The VLC based fingerprinting positioning technique proposed in [39] addressed this problem by using a Bayesian model in which the possibility of disturbances is taken into account. By using such model, the proposed algorithm is robust to obstacles that block the line of sight between LEDs and the mobile device. The simulation result showed that an error distance of 0.81 m in an area measuring 30 × 30 m${}^{2}$ can be achieved with this method.

#### 3.2.2. *k*-Nearest Neighbors (*k*-NN)

*k*-NN is a simple algorithm that can be used for estimating the positions of mobile device with fingerprinting. Assume that $S_{ij}$ is the received signal strength from the *i*th base station measured at the *j*th location candidate during the offline phase, and $s_{i}$ is the online measured signal strength from the *i*th base station, where i=1,2,…,m and j=1,2,…,n. The distance $d_{j}$ between the online measured signal strength vector $s=(s_{1},s_{2},\ldots ,s_{m})$ and the offline signal strength vector $S_{j}=\left(S_{1j},S_{2j},\ldots ,S_{mj}\right)$ measured at the *j*th location candidate is calculated as [38]:

(5) $$\begin{array}{c} d_{j}=\sqrt{\sum_{i=1}^{m}{\left(s_{i}-S_{ij}\right)}^{2}},j=1,2,\ldots n \end{array}$$

After calculating the distances with respect to all location candidates, *k* location candidates having smallest distances are chosen and the coordinate $\left(\hat{x},\hat{y}\right)$ of the mobile device can be calculated as [38]:

(6) $$\begin{array}{c} \left(\hat{x},\hat{y}\right)=\frac{1}{k}\sum_{j=1}^{k}\left(x_{L_{j}},y_{L_{j}}\right) \end{array}$$

where $\left(x_{L_{j}},y_{L_{j}}\right)$ are the coordinates of the *j*th location candidate.

For simplicity, *k* can be set to 1 as in [40]. In these cases, the estimated position of the mobile device cannot be an arbitrary location in the environment but one of the location candidates. Although the mechanism of *k*NN is simpler than the probabilistic method, one can achieve high positioning accuracy with *k*NN by increasing the number of location candidates. An experiment was conducted with the location candidate inter-distance set to 10 cm in a room measuring 180 × 120 cm${}^{2}$ that achieved an average distance error ranging from 15 to 20 cm [40]. Note that in a wider room, the location candidate inter-distance of 10 cm might lead to higher costs for data collection in the offline phase, and longer times for calculating the distances to all location candidates in the online phase. Since signals from different LEDs need to be distinguished for the data collection in both offline and online phases, a mechanism for extracting the signal from different LEDs is required. The authors of [40] used frequency division multiplex to separate signals from different LEDs. Sinusoidal waves were generated to supply the LEDs so that the light emitted from LED had the intensity varying according to these waves. In this way LEDs can transmit signals at different frequencies. Then the power of each modulating frequency will be extracted from the received power spectral density.

Another VLC based fingerprinting positioning system using *k*-nearest neighbors was conceptually proposed in [41] and then detailed in [42]. In this system, the position was determined as the middle between two nearest location candidates (*i.e.*, *k* = 2). The simulation was conducted with four LED base stations in a room measuring 10 m × 9 m × 3.1 m. Space-time block coding was used for multiplexing. In the simulation, walls, windows and many obstacles such as chairs, wooden tables, and a bookcase with different reflectivity were assumed. The mobile device was assumed to move according to a 32-step path. In each step, the peaks of the impulse response time from different LEDs were observed and compared to the fingerprinting map of the impulse response time collected in the offline phase. The results showed that the estimation errors ranged from 0.2 m to 0.8 m.

#### 3.2.3. Correlation

Correlation is a new technique for fingerprinting matching. This technique is based on the idea that the correlation of the mixed received signal from all base stations and the transmitted signal of a specific base station is higher when the mobile device is closer to that base station. The performance of this technique depends on choosing the pattern of the signal transmitted from the base stations so as to yield high correlation with the received signal.

A VLC based positioning system using fingerprinting correlation to determine the location was proposed in [43,44]. The system consists of four LED base stations, named *A*, *B*, *C*, and *D*, that transmit signals with different patterns [0101], [1001], [0110], and [1010] respectively. Assuming that all base stations are synchronized to transmit at the same time, the received signal *R* will be:

(7) $$\begin{array}{c} R=[P_{rB}+P_{rD},P_{rA}+P_{rC},P_{rC}+P_{rD},P_{rA}+P_{rB}] \end{array}$$

where $P_{rA},P_{rB},P_{rC}$, and $P_{rC}$ are received power from the base stations *A*, *B*, *C*, and *D*.

The correlations between the received data and each pattern are:

(8) $$\begin{array}{c} \left\{\begin{array}{c} C_{RA}=2P_{rA}+P_{rB}+P_{rC}\\ C_{RB}=P_{rA}+2P_{rB}+P_{rD}\\ C_{RA}=P_{rA}+2P_{rC}+P_{rD}\\ C_{RA}=P_{rB}+P_{rC}+2P_{rD} \end{array}\right. \end{array}$$

The correlation sum ratios for *x* ($CSR_{X}$) and the correlation sum ratios for *y* ($CSR_{Y}$) are calculated as:

(9) $$\begin{array}{c} \left\{\begin{array}{c} CSR_{X}=\frac{C_{RA}+C_{RC}}{C_{RA}+C_{RB}+C_{RC}+C_{RD}}\\ CSR_{Y}=\frac{C_{RA}+C_{RB}}{C_{RA}+C_{RB}+C_{RC}+C_{RD}} \end{array}\right. \end{array}$$

**Figure 4:**
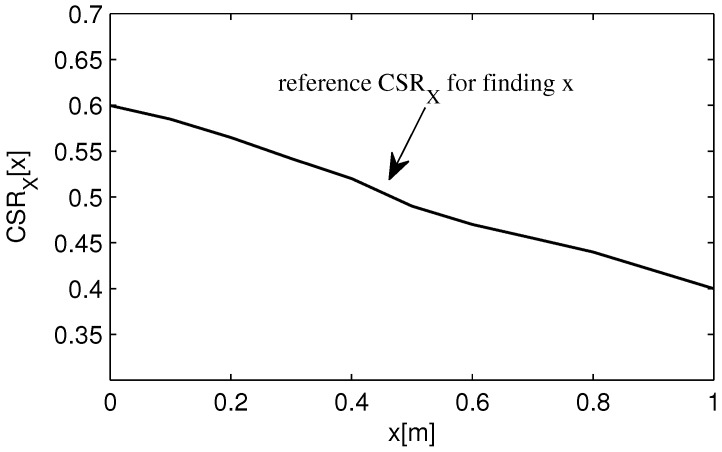
Reference correlation sum ratio for finding x.

For any values (x,y) of the receiver coordinate, the value of $CSR_{X}$ is related only to *x*, independent of *y*, and vice versa for $CSR_{Y}$. During the offline phase, the values of $CSR_{X}$ and $CSR_{Y}$ are collected from all over the environment to create the reference $CSR_{X}$ corresponding to *x* and the reference $CSR_{Y}$ corresponding to *y* as shown in Figure 4. On the online phase, the value of $CSR_{X}$ and $CSR_{Y}$ are calculated and compared to the reference $CSR_{X}$ and $CSR_{Y}$ to find the coordinate (x,y). The experimental was conducted in a space measuring 1×1×1.2 m${}^{3}$ and the result showed that the maximum and mean distance errors were 12.46 and 4.38 cm, respectively.

#### 3.2.4. Type of Signal Features Used for Fingerprinting

Various features of the signal can be chosen for fingerprinting. The accuracy of a fingerprinting algorithm greatly depends on the type of features used. Therefore, fingerprinting algorithms can also be classified based on the type of signal features used for fingerprinting. The most basic type is signal strength, or the signal strength vector type which consists of signal strengths from different base stations. To enhance the accuracy, multiple received signal strengths can be used as fingerprinting. However, there are other types of fingerprinting. In [42], the fingerprinting was the set of impulse response time from multiple base stations. In [43,44], the fingerprinting was based on a series of received signals in a period of time. In [45,46], the fingerprinting was chosen as the so called extinction ratio, which is the ratio between received powers when bit 1 and bit 0 are transmitted. The simulation result showed that an average distance error of 1.58 cm was achieved in a triangular cell measuring 60 cm at each sides.

An array of multiple PDs tilted at different angles to receive the light radiated from a unique LED base station in the room was used in the studies [47,48]. Since the light impinges at different angles incidence, the received power varies at different PDs. In addition, at different position in the room, the distance from the PD array to the LED will be different, and the angle of incidence at each PD will also be different. Therefore, the received power gain caused by the angle can be measured, to be used as fingerprinting for positioning. The simulation result showed that the achieved mean and maximum errors were 4 cm and 13 cm, respectively.

In [49], the fingerprinting was the combination of received signal strength and angle of arrival of the signal. To acquire the angular information, multiple PDs placed at different angles were used to receive the light signal radiated from a unique LED in the room. The received signal strengths at different PDs were compared to estimate the angular information. The positioning accuracy of 6 cm in a space measure 2×2×2 m${}^{3}$ was demonstrated through an experiment.

In [50], the fingerprinting was the combination of the light intensity and more importantly the signal pattern. The light intensity was received by a PD connected to either a smart-phone or a computer. The signal pattern was decoded as a pattern of stripes on the image. LEDs transmitted these signal patterns by blinking at high frequency. Then the camera on a smart phone used the rolling shutter mechanism to receive the transmitted signal. In the offline phase, a map of light intensity and signal pattern was created. In the online phase, a probabilistic approach was used to determine the position of the smart phone.

### 3.3. Triangulation

Triangulation determines the absolute position by using the geometric properties of triangles. There are two derivations of triangulation: lateration and angulation. Lateration techniques, which involve the time of arrival (TOA), the time difference of arrival (TDOA), and the received signal strength (RSS), estimate the position based on the measured distances from the mobile device to multiple LEDs base stations. Angulation, map or angle of arrival (AOA) relies on the measured angles relative to multiple base stations to find the position of the mobile device.

#### 3.3.1. Time of Arrival—TOA

**Figure 5:**
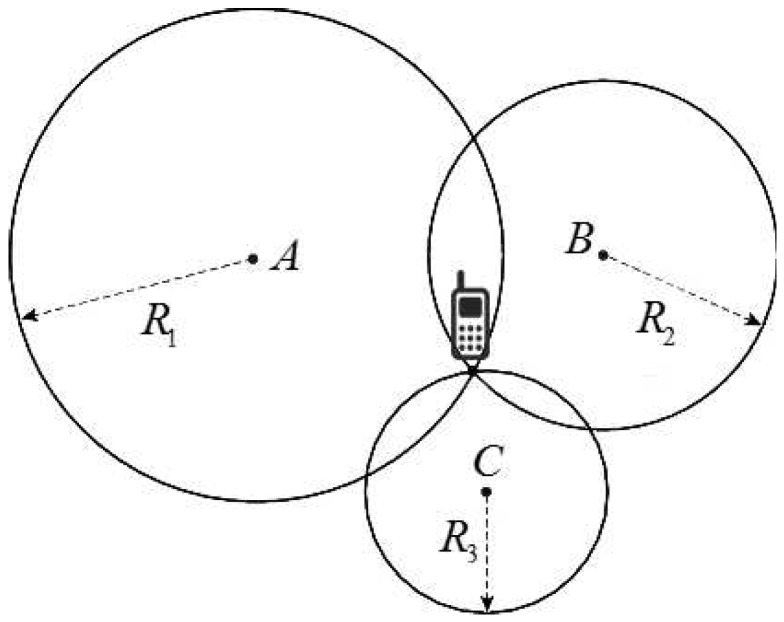
Positioning based on TOA.

TOA, which is the basis of the GPS system, calculates the distances between LEDs and mobile devices from the arrival time of signals and then uses these estimated distances to derive the position of the mobile device. For visible light, the distance is calculated by directly multiplying the propagation delay of the signal by the speed of light. To estimate a 2-D location, distances to at least three base stations must be obtained. In Figure 5, $R_{1}$, $R_{2}$, and $R_{3}$ are the distances to three base stations *A*, *B*, and *C*. The position of the mobile device is determined as the intersection of the three circles with radii of $R_{1}$, $R_{2}$, and $R_{3}$ and centers at *A*, *B*, and *C*.

In practice, the position of the mobile device can be determined using least-squares algorithm. Suppose that (x,y) is the coordinate of the mobile device, $\left(x_{1},y_{1}\right)$, $\left(x_{2},y_{2}\right)$, ..., $\left(x_{n},y_{n}\right)$ are the coordinates of *n* base stations, and $\Delta t_{1}$, $\Delta t_{2}$, ..., $\Delta t_{n}$ are the measured propagation delays of light signals from *n* base stations. The values of *x* and *y* are chosen to minimize the cost function [19]:

(10) $$\begin{array}{c} F\left(x,y\right)=\sum_{i=1}^{n}r_{i}^{2}\left(x,y\right) \end{array}$$

where the residual $r_{i}^{2}\left(x,y\right)$ is given as:

(11) $$\begin{array}{c} r_{i}^{2}\left(x,y\right)=c\Delta t_{i}-\sqrt{{\left(x_{i}-x\right)}^{2}+{\left(y_{i}-y\right)}^{2}} \end{array}$$

where *c* is the speed of light.

In [51], the accuracy of a VLC based positioning system using TOA was analyzed. Orthogonal frequency division multiplexing (OFDM) was used to separate the signals from the different LEDs. The LEDs and the PD receiver were assumed to be perfectly synchronized to a common clock. The theoretical limits on the estimation accuracy calculated by using the Cramer-Rao bound showed that the error ranged from 2 to 6 cm.

#### 3.3.2. Time Difference of Arrival—TDOA

**Figure 6:**
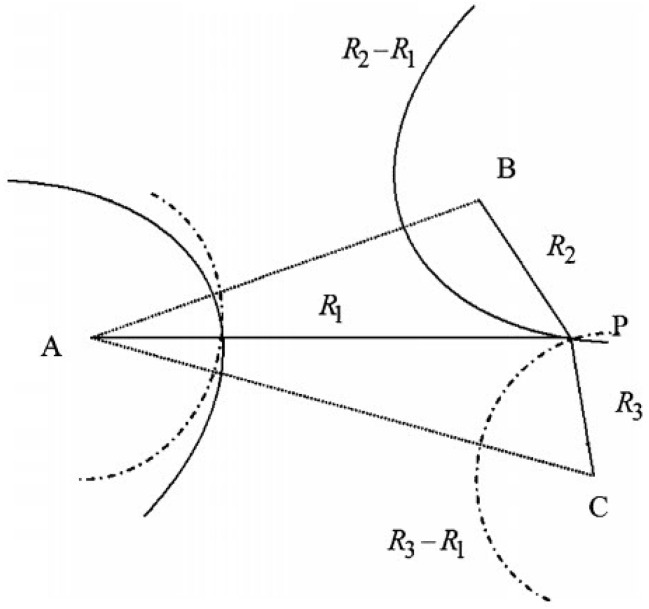
Positioning based on time difference of arrival (TDOA).

Suppose that at the same time all of the LED base stations transmit signals to the mobile device. Due to the difference in the distance from the mobile device to the LEDs, the times at which the signals arrive at the mobile device will be different. TDOA algorithms determine the position of the mobile device based on the time difference of arrival of signals from multiple LEDs. For each TDOA with respect to a pair of base stations, the difference in the distances from the mobile device to the pair can be calculated by multiplying the time difference by the speed of light. Therefore, for each TDOA measurements we can determine a hyperbola of possible positions whose distances to the pair of base stations have a constant difference. To enable 2-D positioning, TDOA measurement with respect to at least three base stations must be obtained. As shown in Figure 6, given two TDOAs with respect to three base station *A*, *B*, and *C*, the two derived distance differences $R_{2}-R_{1}$ and $R_{3}-R_{1}$ define two hyperbolas whose intersection is determined to be the position of the mobile device.

Let (x,y) denote the coordinates of the mobile device and $\left(x_{i},y_{i}\right)$ and $\left(x_{j},y_{j}\right)$ denote the coordinates of the *i*th and *j*th LEDs then the distance difference to the *i*th and *j*th LEDs, denoted by $R_{ij}$, defines a hyperbola as follows:

(12) $$\begin{array}{c} \begin{array}{c} R_{i,j}=\sqrt{{\left(x_{i}-x\right)}^{2}+{\left(y_{i}-y\right)}^{2}}-\sqrt{{\left(x_{j}-x\right)}^{2}+{\left(y_{j}-y\right)}^{2}} \end{array} \end{array}$$

The position of the mobile device is determined as the intersection of these hyperbolas.

In [52,53], a VLC based positioning system used for indoor location based service applications in restaurants, museums, department stores and theaters was proposed. The visible light from four LED panels was modulated using BPSK. After obtaining TDOA information, a nonlinear least square algorithm was used to determine the location. Simulation results showed that the average error was 0.14 m.

In [54,55], a TDOA based indoor positioning system using VLC was proposed. The system consists of a PD receiving signals from multiple LEDs whose positions are known by the receiver. Time division multiplexing was used to separate the signals from different LEDs. Each LED transmits a pilot signal which does not convey any information to the PD. Therefore, the PD has no way to know the ID of the received signal. After receiving a series of anonymous pilot signals from multiple LEDs, a special guessing mechanism was used to find the IDs of the all signals and the position of the mobile device was determined. Simulation results showed that the average error was 3 cm.

In [56] an approach similar to that of [54,55] was applied but a sinusoidal pilot signal was used instead of a square pulse. A simulation was conducted that included consideration of the rising and falling times of the LED. The result showed that the average error distance was 68.2 cm.

With the TDOA algorithm, the important part is estimating the time difference or the distance difference of arrival. There are many methods for extracting the TDOA from the received signal. In [54,55,56], since time division multiplexing was used, the signals from different base stations were separated and thus the TDOA can be obtained easily. In [57,58,59,60], frequency division multiplexing was used, with which the signals from different base stations were mixed together, and TDOA was estimated based on phase difference of arrival.

In [57] a VLC based positioning system for automobiles was presented. The two taillights in the vehicle ahead transmit an intensity modulated sinusoidal light signal to two receivers in the vehicle behind. The light signals from the different taillights were modulated at different frequencies. Bandpass filtering was applied to separate the two signals from the mixed signal at each receiver. After that, the phase difference of arrival of signals at each receiver was determined. The distance difference then can be calculated easily based on the phase difference, the known modulated frequency and the speed of light. The system was tested with a simplistic hardware at the range of 1 m and an accuracy of 1 cm was achieved.

The indoor positioning system proposed in [58] also used the same mechanism of modulation and multiplexing as in [57]. In the first step, the sinusoid light signals from three LED base stations located in the ceiling were modulated at different frequencies. At the receiver, a bandpass filter whose pass band was adjusted to the frequency of each base station was used to extract signals from the different base stations. A Hilbert transform was used to extract the in-phase and quadrature components of each signal, to calculate the phase differences of each pair of signals. In the second step, the modulated frequencies were switched between base stations. After the same process, the phase differences were calculated again. Based on the calculated phase differences, the distances from the receiver to the three base stations were calculated and the position was determined easily using trilateration. Without any noise assumed, the simulation reported a very high accuracy of less than 1 cm achieved by this system.

The authors of [59] extended the work in [58] by adding additive white Gaussian noise to the simulation and applying statistical methods to minimize the estimation error after the positioning stage. The simulation showed that the proposed statistical method can reduce the estimation error from 15.3 cm to less than 2 cm in a room space of 5 m × 5 m × 3 m.

In [60], light signals from multiple base stations were modulated to transmit in sinusoidal form. Based on measuring the peak-to-peak amplitude of the received sinusoid light signals, the phase differences were calculated. Then, based on the phase differences, the known modulation frequency and the speed of light, the TDOA were obtained to determine the position.

**Figure 7:**
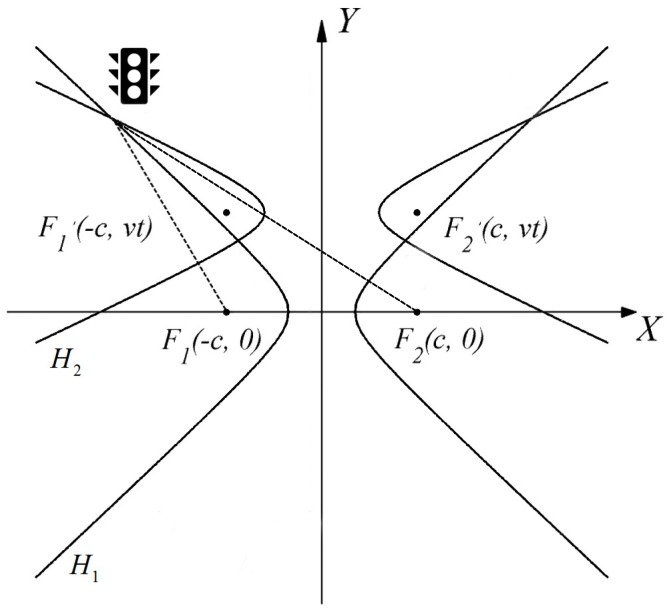
TDOA with one traffic light and two PDs.

Usually TDOA based methods require at least three base stations or three receivers to estimate the position. In [61], a clever method was proposed to enable the 2-D positioning with only one traffic light transmitting signal containing its position information to the two PDs mounted in the front of the vehicle. The proposed method is described in Figure 7. At time $t_{1}$, the signal transmit from a traffic light is received by the two PDs and the TDOA $\Delta t_{1}$ along with the corresponding hyperbola $H_{1}$ are obtained. At time $t_{2}$ when the vehicle is closer to the traffic light, the second hyperbola $H_{2}$ corresponding to the TDOA $\Delta t_{2}$ is obtained. Assuming that the displacement of the vehicle from $t_{1}$ to $t_{2}$ is known, the position of the traffic light relative to the vehicle is determined as the intersection of the two hyperbolas. Finally the absolute position of the vehicle is calculated based on the relative and absolute position of the traffic light. The simulation results showed that the accuracy of this system greatly depends on the distance between the vehicle and the traffic light, as well as the speed of the vehicle. More specifically, the accuracy increased when the distance increased. For example, the positioning error was very high when the distance was shorter than 5 m, but when the distance was 50 m, the positioning error was only 0.5 m. Regarding the speed of vehicle, as one might expect, the positioning error increased when the speed increased. However when the distance was greater than 20 m, the speed of vehicle had a very small effect on the accuracy.

#### 3.3.3. Received Signal Strength—RSS

**Figure 8:**
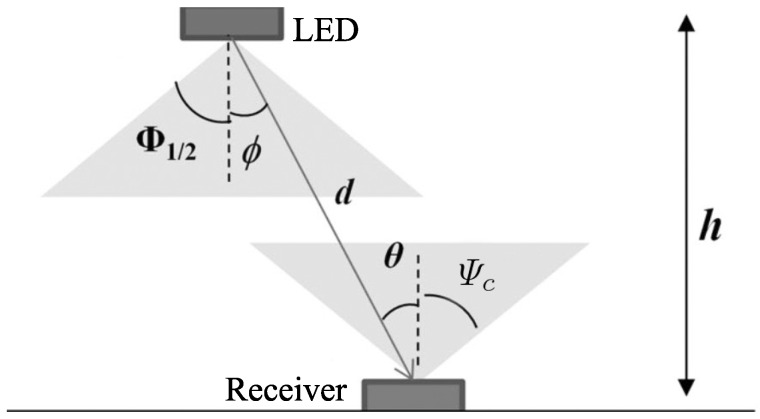
Light transmission between LED and receiver.

RSS algorithm determines the position of the mobile device based on the received signal strength. The light path from the LED to the receiver is illustrated in Figure 8.

Suppose that LED has a Lambertian radiation pattern, the received power can be calculated as [62]:

(13) $$\begin{array}{c} P_{r}=\left\{\begin{array}{c} \begin{array}{c} P_{t}\frac{(m+1)A}{2\pi d^{2}}\cos^{m}\left(\phi \right)T_{s}\left(\psi \right)g\left(\psi \right)\cos\left(\psi \right)\\ \;\;\;\;\;\;\;\;\;\;\;\;\;\;\;\;\;\;\;\;\;\;\;\;\;\;\;\;\;\;\;\;\;\;\;\;\;\;\;\;0\leq \psi \leq \Psi_{c} \end{array}\\ 0,\;\;\;\;\;\;\;\;\;\;\;\;\;\;\;\;\;\psi >\Psi_{c} \end{array}\right. \end{array}$$

where $P_{t}$ is the transmitted power, *A* is the physical area of the detector in the PD, *m* is the order of Lambertian emission, *φ* is the irradiance angle, *ψ* is the incidence angle, $T_{s}\left(\psi \right)$ is the gain of the optical filter, g(ψ) is the gain of the optical concentrator, $\Psi_{c}$ is the field of view at the PD, and *d* is the distance between the LED and receiver.

In theory, given that all other parameters are known, the distance can be derived from the received power using Equation (13). Then trilateration is used to determine the location similarly to the TOA technique. In practice, however, the light propagation suffers from multipath, reflection, obstruction, and many other factors which make the result obtained from Equation (13) unreliable. For example [62] examined the effect of the reflected light on the accuracy of a VLC based positioning system using RSS. In that system, the distance from the mobile device to the LED base stations were derived from the received power using Equation (13) directly. The simulation results showed that the root mean square error increased from 4 cm, which was the result with no reflected light, to 80 cm when the reflected light was considered. Therefore, the main challenge when applying RSS is to find an appropriate path loss model to estimate the distance.

A VLC based positioning system using RSS was proposed in [63,64,65,66]. The visible light from three LED base stations installed in the ceiling was modulated in Quadrature Phase Shift Keying (QPSK) at different frequencies. After receiving the mixed signal, the signals from the different LEDs were extracted and the distances to each LED was calculated in two steps. In the first step, since the system did not use any type of inertial sensor (e.g., acceleration sensor, gyroscope) to obtain the information about the orientation of mobile device, the rough distance was calculated without considering the effect of irradiance angle and incidence angle on the received power. Because the light signal was attenuated by the effect of these two angles, the roughly-estimated distance was longer than the real distance. In the second step, the maximum possible distance was calculated based on the known height of the ceiling and the illuminating coverage of the LED. Then a compensated distance was calculated based on the maximum possible distance and the roughly estimated distance. The final estimated distance was calculated by subtracting the compensated distance from the roughly estimated distance. The location then is determined using trilateration and the linear least square method. The experiment was conducted in a space of 60 cm×60 cm×85 cm and an error of 6 cm was reported.

In [67,68] a gyroscope was used to obtain the orientation information of the mobile device. They found that the estimated position given by RSS positioning tend to move toward the direction of a tilting receiver. A compensation mechanism for the error caused by the tilt angle of the receiver was proposed. The experiment showed that by applying the proposed compensation, the average distance error was reduced from 40 cm to 1.5 cm.

While systems in [63,64,65,66,67,68] used multiple base stations transmitting signals to a single receiver, in [69,70], three PD receivers installed on a disk of radius 20 cm on top of the mobile device were used to receive a signal from a single LED base station. The distances from the LED to the receivers were estimated from the received signal strengths at each receiver. The position of the mobile device was determined based on the known coordinates of the LED, the arrangement of multiple PDs (*i.e.*, their relative position to each other), and the distances from the LED to multiple PDs. The simulation showed that a distance error of less than 1.5 cm in a 2×2×2 m${}^{3}$ environment was achieved. Note that the system aimed to autonomous machine control application such as cleaning robots. Therefore, the size of 40 cm in diameter of the receiver is acceptable. However, for applications in which the sizes of the mobile devices are limited, this technique is almost impractical.

In [71], optical orthogonal code (OOC) was used to distinguish multiple signals. Then the distances to different base stations were calculated based on RSS, and trilateration was used to determine the position. The simulation showed that the average error distance was 8 cm in an area of 12 m × 35 m.

A creative technique using dual-tone multi-frequency (DTMF), which is widely used in telephone dialing, voice mail, and electronic banking systems to separate the light signal from different LED base stations was proposed in [72]. The paper also developed a new algorithm for calculating the path loss based on the RSS corresponding to different frequencies. The coordinates of LED base stations were assumed to be known to the mobile device. A simulation in an area of 2 m × 2 m reported an average error distance of 18 mm.

In [73], beacon light modulated in Binary Frequency Shift Keying (BFSK) transmitted a signal containing coordinates of LED base stations to the mobile device. The duty cycle remained the same at different frequencies to prevent flickering. Random channel hoping was used to avoid persistent collision among light sources. Each LED base station transmitted beacon signals at a random channel for a certain period, and then hopped to other channels. The RSS was translated to distance using a simple model which assumed that all LEDs were facing downward and that the receiver was facing squarely upward toward the ceiling. Then, the least mean square (LMS) method was used to determine the position. An experiment was conducted in an empty conference room of 5 m × 8 m and a cubicle area of 3.5 m × 6.5 m. The accuracy reported was 0.3 m and 0.7 m for the conference room and the cubicle area, respectively.

The work in [73] was extended in [74] by addressing more practical challenges including enabling reliable communication and robust localization in case of insufficient light sources or imperfect orientation. The simulation results showed that an accuracy of 0.4 m in a space 20 m × 20 m × 3 m was achieved.

In [75], the receiver calculated the light intensity at each position based on its knowledge about the system. Upon receiving the light from the LED, the received light intensity was compared to the pre-calculated light to find the position of the mobile device. The simulation showed that depending on the angle of the mobile device, distance errors of 3 to 35 cm were achieved.

A positioning system for moving robot using RSS was demonstrated [76]. Multiple LED base stations transmitted their IDs to the PD mounted on the robot. TDM was used to separate different light signals. A method called bit stuffing was proposed to prevent interferences between ID pulses and thus allowed a simple function to be used for determining the position. The experiment was conducted in a space 120 cm × 120 cm × 170 cm. The maximum and mean error distances were reported to be 10.3 cm and 3.2 cm, respectively.

The system in [77] also used RSS and TDM. The light was modulated in On-Off Keying (OOK) to transmit the coordinates of LED base stations. A frame structure was designed to keep the transmission power stable while different bits were transmitted. Based on this frame structure, an algorithm was developed to calculate the path loss. The simulation results showed that depending on the noise level, the average error distance ranged from 0.5 mm to 7.3 mm in a space of 3 m × 3 m × 3 m.

A state estimation model which was inspired by Kalman Filter recursive estimation to achieve localization and motion tracking was proposed in [78]. There were two scenarios in the estimation process. In the first scenario, the RSS was measured when the receiver was directed perpendicular to the ceiling and the state (*i.e.*, the position) was estimated. In the second scenario, RSS was measured and the effect of the rotation of the receiver was added to the state model. Then the state was updated corresponding to the changes in the state model. Simulation results showed that position and velocity errors of 5 cm and 10 cm/s were achieved in a space of 6 m × 6 m × 4 m.

A Cramer-Rao bound was used in [79] to analyze the theoretical accuracy of an indoor VLC based positioning system using RSS. Signals from different base stations were separated by frequency. The cases where LEDs and PD are not parallel were discussed. The positioning accuracy corresponding to two types of LED array layouts: square and triangle were analyzed. The results showed that the theoretical accuracy corresponding to the triangle LED layout was higher than that of the square LED layout. In addition, the accuracy of 4.78 cm was shown to be achievable with specific parameter values.

An idea of measuring RSS by calculating the cross correlation between received signal and transmitted signal was proposed in [80]. The effects of the irradiance and incidence angle of the light on the RSS were compensated for by multiplying the RSS calculated by cross correlation with a weighting factor. Trilateration and the linear least square method were used to determine the position. The experiment was conducted with three LED base stations transmitting orthogonal codes modulated in OOK to a PD moving in a plane parallel to that of the LEDs at a distance of 2 m. The average error distance reported was 6 cm.

In [81] a VLC based three dimensional indoor positioning using RSS was proposed. To reduce the error of the RSS-to-distance calculation, a compensated distance was included in the path loss model. Using the proposed scheme, multiple LED base stations were utilized to obtain higher positioning accuracy. Simulation results showed that the accuracy was increased when the number of LED base stations was increased.

#### 3.3.4. Received Signal Strength Ratio and Received Signal Strength Difference

Instead of directly translating the received power to the distance, the received signal strength ratio (RSSR) technique translates the ratio of the received power from multiple base stations to the ratio of distance and determines the position based on the distance ratio. The advantage of RSSR compared to RSS is that the ratio of received power would cancel the error caused by the non-zero irradiance angle of the light. Therefore, the ratio of received power can be translated into the distance ratio with less error than the distance directly translated from the power, thus achieving higher accuracy. The limitation of this technique is that the surface of the receiver must be parallel to the LED, which would be impractical for some applications.

RSSR indoor positioning systems based VLC were proposed in [82,83,84]. In the proposed systems, the receiver was assumed to be parallel to the LED and thus the incidence angle and the irradiance angle are equal. Also, the incidence angle was assumed to be smaller than the FOV at all measured points. Recalling Figure 8 and Equation (13), the received power in this case can be rewritten as:

(14) $$\begin{array}{c} P_{r}=P_{t}\frac{(m+1)A}{2\pi d^{2}}\cos^{m}\left(\phi \right)=\frac{K}{d^{m+3}} \end{array}$$

Where *K* is a value independent of the receiver position.

From Equation (14), the distance ratio can be calculated easily from the RSSR:

(15) $$\begin{array}{c} \frac{d_{2}}{d_{1}}=\sqrt[{n+3}]{\frac{P_{r1}}{P_{r2}}} \end{array}$$

Supposing that $\left(x_{1},y_{1},h\right)$ and $\left(x_{2},y_{2},h\right)$ are the coordinates of the two LED base stations, the following equation is given:

(16) $$\begin{array}{c} \frac{\sqrt{{\left(x-x_{2}\right)}^{2}+{\left(y-y_{2}\right)}^{2}+h^{2}}}{\sqrt{{\left(x-x_{1}\right)}^{2}+{\left(y-y_{1}\right)}^{2}+h^{2}}}=\frac{d_{2}}{d_{1}} \end{array}$$

Therefore, given an RSSR, a circle or a perpendicular bisector (in case d2d1 = 1) is defined. The position of the receiver then is determined as the intersection of these circles of bisectors.

In [82,83,84], time division multiplexing was used to distinguish signals from different LEDs. The experiment was conducted in an area measuring 1×1 m${}^{2}$ and distance error ranging from 3.89 to 1.68 cm were reported.

Another derivation of RSS is the received signal strength difference technique, which was proposed in [85,86]. The distance was not derived directly from the received power but from the difference in the received power between logical 0s and 1s. The IDs of base stations were transmitted to the receiver through visible LED light modulated in OOK format. To lessen the demand of perfect synchronization between base stations, the framed slotted ALOHA (FSA) protocol was used for the LED base stations to transmit their IDs without collisions. The difference in the received power of the logical 0 and 1 was calculated and translated to the distance using a proposed model. After that, the linear least square method was used to solve trilateration equations to obtain the position. The simulation reported a distance error of 11 cm (no direct sunlight exposure assumed) and 17 cm (direct sunlight exposure assumed) in an area measuring 6 m × 6 m, achieved by this method.

#### 3.3.5. Angle of Arrival—AoA

**Figure 9:**
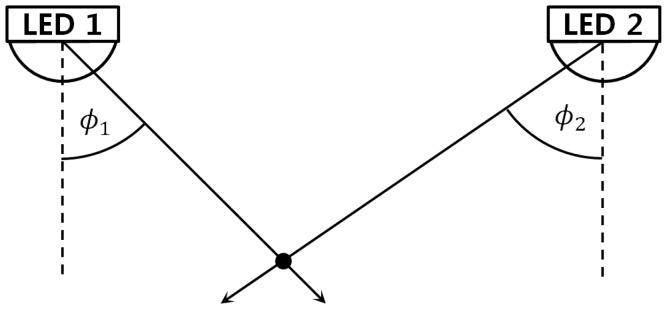
Positioning based on angle of arrival (AOA).

AOA algorithms base on the estimated angles of arrival of signals from multiple LEDs to determine the position of the mobile device. The mechanism of AOA is described in Figure 9. After obtaining the angle of arrival, the position of the mobile device is determined as the intersection of multiple bearings.

Compared to an RF based system, a VLC based positioning system has a big advantage using AOA since the signal in VLC is transmitted in line of sight (LOS). Compared to other techniques used in VLC based positioning, AOA has the advantage that the position of the mobile device can be obtained with three measurements for 3-D positioning or two measurements for 2-D positioning. Another advantage is that AOA does not require the synchronization between LEDs.

Usually, to obtain the angle of arrival, a PD array can be used. Since the light radiated from a LED follows the Lambert cosine law, the changes in the angle of arrival will lead to changes in the received power at predictable manners. Therefore, the angle of the light can be inferred from the difference between the received power at a known angle and the received power at the current angle.

**Figure 10:**
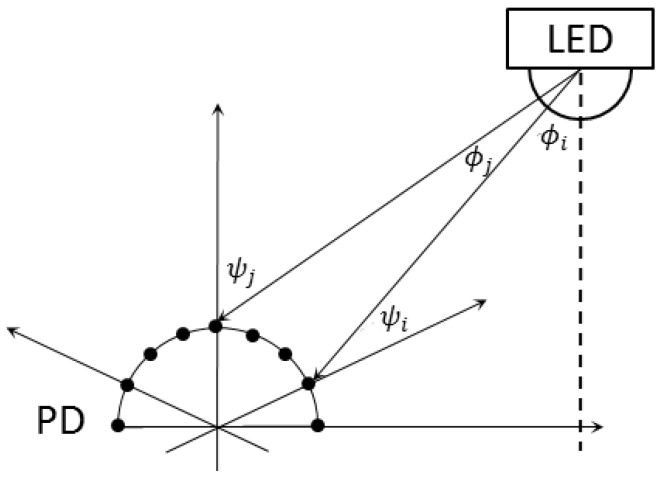
Circular PD array for estimating AOA.

In [87], a circular PD array was used to estimate the AOA. This PD array is illustrated in Figure 10. As expressed by Equation (13), the light signal power received by each PD in the PD array is affected by the distance *d*, radiance angle *φ*, and the incidence angle *ψ*. The distance *d* and the radiance angle *φ* can be assumed to be the same for all PDs since the size of the PD is very small compared to the distance. Therefore, the received power is determined only by the incidence angle *ψ*, which is different at each PD due to the circular placement as shown in Figure 10. The irradiance angle of the light was determined using a proposed truncated-weighting algorithm, which is a weighted sum of angles of PDs in the PD array. The weight for each PD angle was determined by comparing the received power at different PDs. The simulation result showed that the distance error ranged from 5 to 30 cm.

Using an accelerometer, [88,89] could determine the AOA with only one PD. To do that, the PD was rotated to different orientations and the received power corresponding to these orientations was measure. Note that the position of PD must be the same while being rotated. By using an accelerometer, the orientation of the PD is always known. For each LED base station, two measurements corresponding to two orientations of the PD were made. Based on the two received powers measured at two orientations, the irradiance of the light was obtained. The locations of the LED base stations were transmitted through the VLC signal. Multiple signals were separated using TDM. After three irradiance angles corresponding to three LEDs were obtained, the position of the mobile device was determined. The simulation showed that the average position error was less than 25 cm, given that the receiver is static.

A subsequent paper [90] improved upon the work of [88,89] by using multiple tilted PDs combined with an accelerometer to estimate the AOA without the need for rotating the mobile device. The AOA estimation can be performed faster and thus user mobility is supported in this system. To improve the accuracy, a power correction method was proposed to mitigate the error which is caused by the non-zero distances between multiple PDs. The experiment was conducted with three LEDs mounted on the ceiling and 4 PDs grouped into a pyramid structure. The TDM was used to separate the signals from multiple LEDs. The result showed that the mean position error was less than 6 cm even when the receiver moved at the average speed of 1.3 m/s.

One of the first studies on VLC based positioning [91] did not use LEDs, but fluorescent lights to determine the position. A single PD was used to estimate the vertical and horizontal angles between the PD and the fluorescent lamps. This was possible since the angle of view of the PD is small, $10^{\circ }$. Therefore, when the PD was directed toward the fluorescent lamps, by considering the height of the ceiling where the lamps were installed and the size of the lamps, the angles and the position of the receiver could be estimated. The experiment was conducted in a wide area with 22 fluorescent lights and an average distance error of tens of cm was reported.

A more sophisticated method for estimating the AOA was presented in [92]. A group of three orthogonal PDs was used to receive light signals from multiple LED base stations. The signals were modulated with distinct frequency channels, between 2 kHz and 3 kHz, so that they can be separated in the mixed received signal. Since the angles of the PDs are different, they were preferentially sensitive to light incident along different directions. The difference in the received power at these PDs was expressed as functions of the PD angles and light incidence angles. These functions were difficult to solve algebraically since they were piecewise. However, least-angle regression could be used to solve these equations to obtain the incident angle. Simulation results showed that the positioning error of this method was 5 cm.

Then [93] presented a realistic application for VLC based positioning with which the position information was used to align the VLC transmitter and receiver to achieve higher communication SNR. In this system, a single PD was used to obtain AOA information. The PD was assumed to be perpendicular to the floor, which meant that the irradiance and incidence angles were equal. Then the received power at a certain position was expressed as a function of the maximum received power and the irradiance angle. The multiple reflections were also taken into account by adding a path loss exponent to the equation for the received power. The maximum received power of an LED base station was measured beforehand and assumed to be known by the receiver. Therefore, the irradiance angle of an LED can be determined from the received power. The signals from multiple LED base stations were separated by using OFDM. Upon receiving signals from three or more LED base stations, the position can be determined.

### 3.4. Vision Analysis

#### 3.4.1. Basis of Vision Analysis

**Figure 11:**
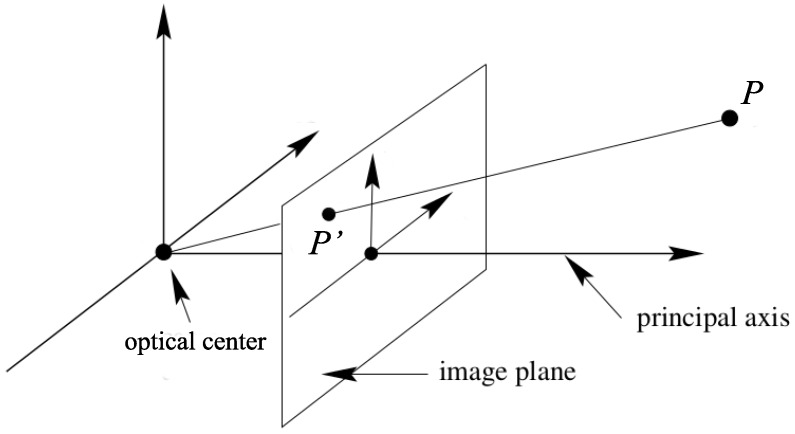
Pinhole camera model.

Vision analysis involves the geometric relations between the 3D positions of objects in the real world and their 2D positions in their projections on the image sensor. All of the geometric relations in vision analysis are derived from the pinhole camera model illustrated in Figure 11.

As illustrated in Figure 11, there are three coordinate systems including the 3D world coordinate system, the 3D camera coordinate system, and the 2D image coordinate system. Suppose that $\left(x^{\prime },y^{\prime },z^{\prime }\right)$ is the coordinate of the point *P* in the camera coordinates and (X,Y,Z) is its coordinate in the world coordinate system. Then (X,Y,Z) can be transformed to $\left(x^{\prime },y^{\prime },z^{\prime }\right)$ through a unique 4×4 matrix [94]:

(17) $$\begin{array}{c} {\left(x^{\prime },y^{\prime },z^{\prime },1\right)}^{T}=\left(\begin{array}{cc} R & t\\ 0 & 1 \end{array}\right){\left(X,Y,Z,1\right)}^{T} \end{array}$$

where *R* is a 3×3 rotation matrix and *t* is a 3×1 translation vector.

Referring to the pinhole camera model, if a point $P^{\prime }\left(x,y\right)$ on the image sensor is the projection of point *P* , then the following relation holds:

(18) $$\begin{array}{c} {\left(x,y,1\right)}^{T}=C{\left(x^{\prime },y^{\prime },z^{\prime },1\right)}^{T} \end{array}$$

where *C* is the camera calibration matrix given as:

(19) $$\begin{array}{c} C=\left(\begin{array}{c} \begin{array}{cccc} f & 0 & 0 & 0 \end{array}\\ \begin{array}{cccc} 0 & f & 0 & 0 \end{array}\\ \begin{array}{cccc} 0 & 0 & 1 & 0 \end{array} \end{array}\right) \end{array}$$

and *f* is the focal length of the lens.

The geometric relation between the 3D world coordinates of a point in the real world and the 2D image coordinates of its projection on the image sensor is described as [94]:

(20) $$\begin{array}{c} {\left(x,y,1\right)}^{T}=C\left(\begin{array}{cc} R & t\\ 0 & 1 \end{array}\right){\left(X,Y,Z,1\right)}^{T} \end{array}$$

Equation (20) is also the basis for the traditional pose and position estimation system used in robot vision or augmented reality applications. In Equation (20), the 2D image coordinate (x,y) and the 3D world coordinate (X,Y,Z) are called reference information. To estimate the pose, that is, the orientation or the position of the camera, multiple sets of (x,y) and (X,Y,Z) must be known.

In the traditional pose and position estimation system, obtaining the reference information is the main challenging problem which explains why these systems are mainly classified based on the manner how reference information is obtained.

With VLC based positioning systems using image sensors, obtaining the reference information is a much easier task. The LED base stations are assumed to be always available in the scene. The 2D image coordinate (x,y) can be easily obtained through image processing thanks to the fast blinking feature of the LED which makes it rare in the natural world. In addition, the 3D coordinates (X,Y,Z) of the LED base stations are obtained through the modulated light signal from the LEDs. Therefore, VLC based vision analysis positioning techniques are classified based on how the geometric relation in Equation (20) is handled with specific equations to solve for determining the position.

In [95], several applications including pose, position and range estimation the using VLC and image sensor were introduced. Regarding pose estimation, the principle for estimating the pose of a single camera based on multiple LEDs was presented. Regarding positioning, a very basic mechanism for determining the position of a moving target using multiple cameras was introduced. In this section, the algorithms for determining the positioning of a mobile device using multiple cameras and VLC will be presented in more detail. We divide these algorithms into two types: single view geometry and vision triangulation.

#### 3.4.2. Single View Geometry

**Figure 12:**
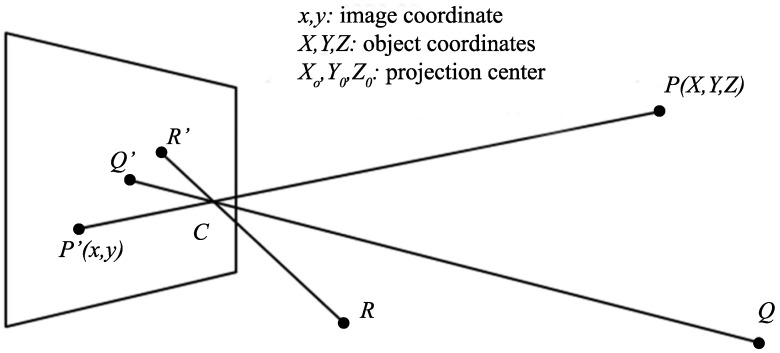
Collinearity condition.

The single-view geometry techniques use a single camera to capture the image of multiple LEDs, as illustrated in Figure 12. Then the geometric relation between the 3D world coordinates, usually obtained from the location information sent by LEDs, and the 2D image coordinates obtained through image processing are used for deriving the position of the camera.

ByteLight [96] has filed a patent for a VLC based positioning system using a camera in a smart phone. The rollight shutter of camera was used to receive the identification codes transmitted from LEDs. These identification codes were associated with specific location information stored in a database. When receiving a single identification code, a smart phone can search its position information from the database. If there were three or more LEDs in the view of the smart phone, single view geometry were used to determine the exact position of the smart phone.

In [96], the details of steps in deriving the position of the smart phone were not revealed. In the literatures, there are usually two methods for deriving the position of the camera: using collinearity condition and using scaling factor.

##### Method of Using Collinearity Condition

Each set of reference information including the 2D image coordinate (x,y) and the 3D world coordinate (X,Y,Z) corresponding to one LED base station creates a set of two collinearity equations, which are the explicit representations of the geometric relations in Equation (20) [97]:

(21) $$\begin{array}{c} \left\{\begin{array}{c} x=-f\frac{R_{11}\left(X-X_{0}\right)+R_{12}\left(Y-Y_{0}\right)+R_{13}\left(Z-Z_{0}\right)}{R_{31}\left(X-X_{0}\right)+R_{32}\left(Y-Y_{0}\right)+R_{33}\left(Z-Z_{0}\right)}\\ y=-f\frac{R_{21}\left(X-X_{0}\right)+R_{22}\left(Y-Y_{0}\right)+R_{23}\left(Z-Z_{0}\right)}{R_{31}\left(X-X_{0}\right)+R_{32}\left(Y-Y_{0}\right)+R_{33}\left(Z-Z_{0}\right)} \end{array}\right. \end{array}$$

where *f* is the focal length, $\left(X_{0},Y_{0},Z_{0}\right)$ is the world coordinate of the camera projection center, and *R* is the rotation matrix defined as [97]:

(22) $$\begin{array}{c} \left\{\begin{array}{c} R_{11}=\cos\phi \cos\kappa \\ R_{12}=-\cos\phi \cos\kappa \\ R_{13}=\sin\phi \\ R_{21=}\sin\omega \sin\phi \cos\kappa +\cos\omega \sin\kappa \\ R_{22}=-\sin\omega \sin\phi \sin\kappa +\cos\phi \cos\kappa \\ R_{23}=-\sin\omega \cos\phi \\ R_{31=}-\cos\omega \sin\phi \cos\kappa +\sin\omega \sin\kappa \\ R_{32=}\cos\omega \sin\phi \cos\kappa +\sin\omega \sin\kappa \\ R_{33}=\cos\omega \cos\phi \end{array}\right. \end{array}$$

Usually the focal length *f* is a known parameter for a specific system, and the world coordinate of the camera projection center which is considered as the position of the mobile device $\left(X_{0},Y_{0},Z_{0}\right)$ is the parameter that needed to be determined. Since there are six unknown parameters in Equations (21) and (22): $X_{0},Y_{0},Z_{0},\phi ,\omega$ and *κ*, at least three pairs of x,y and (X,Y,Z) reference information must be known to find these values of the unknowns parameters. Various solutions are possible under different assumptions for the parameters in Equations (21) and (22).

In [97], the collinearity condition equations was solved using the least squares method. At least three LED base stations and their projections on the image sensor were required to find the position of the camera. The simulation showed that with a sensor of 1000 × 1000 pixels resolution used, a distance error of about 7 cm was achieved.

The approach taken in [98] used a fish-eye lens to take pictures of multiple LED base stations in the same image. With at least three LEDs detected, the collinearity condition equations were solved using the nonlinear least square Levenberg-Marquardt algorithm to estimate the position. Since the fish-eye lens introduces a lot of distortion in the image, an calibration process must be used to correct the image before applying the positioning algorithm. The experiment showed that the maximum horizontal error of the system was less than 10 cm.

In [99], an acceleration sensor was used to obtain information about the orientation of the camera, which is defined by ϕ,ω and *κ*. Since only three parameters, which are $X_{0},Y_{0}$ and $Z_{0}$ in Equation (21) are unknown, the system can determine the camera position with two or more LED base stations captured in the image.

##### Method of Using a Scaling Factor

With the single view geometry approach, using collinearity condition equations might be the most straightforward way to obtain the position of the camera. Given the world coordinates of mobile stations, one just needs to process the image to obtain the image coordinates of base stations and formulate collinearity equations like Equation (21). When the number of equations is sufficient, the world coordinate of the camera can be determined within one step. Some studies, however, might convert the geometric relations into other representations and use them to determine the position of the camera through a number of steps.

For example, [100,101] determined the world coordinate of the camera in two steps. Firstly, the camera coordinates $\left(x^{\prime },y^{\prime },z^{\prime }\right)$ of base stations were determined based on scaling factors. Then the equations of the camera transformation were solved to determine the world coordinate of the camera.

Suppose a point has the world coordinates (X,Y,Z), camera coordinates $\left(x^{\prime },y^{\prime },z^{\prime }\right)$, and image coordinates (x,y). A scaling factor *k* expresses the relation between the camera coordinates and the image coordinates as:

(23) $$\begin{array}{c} \left(x^{\prime },y^{\prime },z^{\prime }\right)=k\left(x,y,f\right) \end{array}$$

where *f* is the focal length of the lens.

Suppose that $\left(X_{1},Y_{1},Z_{1}\right)$, $\left(x_{1}^{\prime },y_{1}^{\prime },z_{1}^{\prime }\right)$ and $\left(x_{1},y_{1}\right)$ are respectively the world coordinate, the camera coordinate, and the image coordinate of point *P*. Similarly, $\left(X_{2},Y_{2},Z_{2}\right)$, $\left(x_{2}^{\prime },y_{2}^{\prime },z_{2}^{\prime }\right)$ and $\left(x_{2},y_{2}\right)$ are respectively the world coordinate, camera coordinate, and image coordinate of *Q*. The pairwise distance between *P* and *Q* , denoted as $d_{PQ}^{2}$, can be written as follows:

(24) $$\begin{array}{c} \begin{array}{c} d_{PQ}^{2}={\left(x_{1}^{\prime }-x_{2}^{\prime }\right)}^{2}+{\left(y_{1}^{\prime }-y_{2}^{\prime }\right)}^{2}+{\left(z_{1}^{\prime }-z_{2}^{\prime }\right)}^{2}\\ ={\left(k_{1}x_{1}-k_{2}x_{2}\right)}^{2}+{\left(k_{1}y_{1}-k_{2}y_{2}\right)}^{2}+{\left(k_{1}f-k_{2}f\right)}^{2}\\ ={\left(X_{1}-X_{2}\right)}^{2}+{\left(Y_{1}-Y_{2}\right)}^{2}+{\left(Z_{1}-Z_{2}\right)}^{2} \end{array} \end{array}$$

With three or more equations like Equation (24), the scaling factors can be found and then the camera coordinates of base stations are determined based on Equation (23).

From camera coordinates and world coordinates of base stations, multiple camera transformation equations like Equation (17) are formulated. Then the least squares method can be used to determine unknown parameters, including the translation vector *t*, which is also the world coordinate of the camera.

In [100], multiple LED base stations transmitted their world coordinates to the camera in a smart phone. The signals from different LEDs were modulated in OOK at different frequency. Then the rolling shutter effect of the camera was utilized to receive the light signal. The experiment results showed that the accuracy was within 10 cm at 90% of the tested locations.

A light-weight indoor positioning system for a wearable device was proposed in [101]. Using the proposed polarization based modulation, a light signal can be modulated at low pulse rate without causing flicker. Therefore, any illuminating light sources, not necessarily LEDs, can be used for transmitting signals. In the experiment, fluorescent lamps were used to transmit beacon signals containing their world coordinates. The camera on an Android smart-phone was used as the receiver. Such necessary information as the focal length and the size of the image sensor was obtained from the EXIF of the image. The world coordinate of the camera, which is also the position of the smart phone, was determined based on a geometric scaling factor. The results showed that the distance errors of less than 30 cm were obtained in 90% of the test cases.

#### 3.4.3. Vision Triangulation

**Figure 13:**
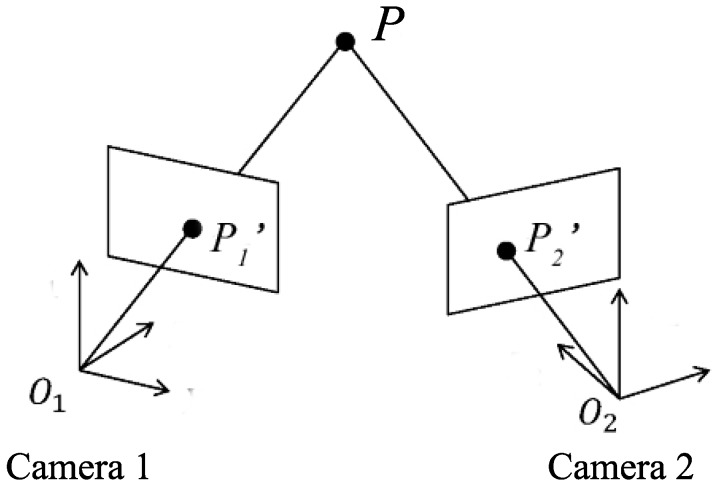
Two-view geometry.

Vision triangulation is a method for 3D position measurement of an object in the real world with multiple known cameras as illustrated in Figure 13.

Suppose that $P_{1}^{\prime }$ and $P_{2}^{\prime }$ are the projections of a 3D point *P* on two cameras, the relation between the world coordinate of *P* and the two image coordinates of $P_{1}^{\prime }$ and $P_{2}^{\prime }$ is mathematically described as [95]:

(25) $$\begin{array}{c} \left\{\begin{array}{c} {\left(x_{1},y_{1}\right)}^{T}=M_{1}{\left(X,Y,Z\right)}^{T}\\ {\left(x_{2},y_{2}\right)}^{T}=M_{2}{\left(X,Y,Z\right)}^{T} \end{array}\right. \end{array}$$

where $M_{i}$ is a 3×4 perspective projection matrix of $i^{th}$ camera defined as [95]:

(26) $$\begin{array}{c} M_{i}=C_{i}\left(\begin{array}{cc} R_{i} & t_{i}\\ 0 & 1 \end{array}\right) \end{array}$$

Usually in a system the calibration matrix $C_{i}$ of all cameras is the same. The relative position and orientation of one camera to other is assumed to be known and thus the rotation matrix $R_{i}$ and the translation vector *t* can be represented using the parameters of one camera. When Equation (25) is expressed in the form of collinearity conditions as in Equation (21), six parameters need to be found given that the focal length *f* which defines the camera calibration matrix is known. Least squares methods can be used to solve these equations to determine the camera position. Other methods which do not use collinearity conditions are presented in [102,103,104].

In [102,103], two cameras installed in the mobile device are used to estimate the distance from the mobile device to four LED base stations. With four estimated distances, the position of the mobile device is determined using the trilateration method. Simulation results show that a positioning accuracy of 1.5 m is achieved by this system.

An implementation of the system proposed in [102,103] was conducted in [104]. Two webcams attached to a PC were used to take the images of four LEDs installed on the ceiling. The accuracy was improved by using higher resolution sensor and by computing the center of an image in terms of a decimal point pixel to reduce the quantization error. The experiment results show that an accuracy of 0.85 m, a 61% improvement over the previous system, was achieved.

### 3.5. Hybrid Algorithm

In [105] an RSS-TDOA hybrid positioning system using VLC was proposed. The distances from the mobile device to base stations were determined based on RSS. Then the RSS-based distance differences were calculated. The TDOA information was obtained by detecting local highest peaks of the received signal. Then each TDOA was translated into a TDOA-based distance difference. The final distance differences were calculated as the weighted sums of the RSS-based and TDOA-based distance differences. Then Newton method was used to solve the equations formulated from the final distance differences, to obtain the position. The reason behind the weighted sum is that the estimations based on both RSS and TDOA contain errors. While RSS estimation suffers from reflected light, TDOA estimation is more sensitive to the fluctuations of the received power due to noises. Simulation results showed that the accuracy of the position determined by TDOA only was higher than that of RSS. More importantly, choosing proper weights, which were 0.34 for RSS and 0.66 for TDOA, yielded the smallest error distance, which was below 5 mm.

The fingerprinting algorithm proposed in [49] was extended in [106] by using an extended Kalman filter (EKF) algorithm to track the position of a moving robot. An array of PDs was mounted on the robot to obtain both RSS and AOA information. Simulation results showed that the error got smaller when the number of PDs increased. For example, increasing the number of PDs from 8 to 16 reduced the error from 41 cm to 20 cm. The results also showed that adding the EKF reduced the average error and removed the cumulative errors.

In [107], RSS and AOA were used in two phases to calculate the positions of mobile devices in indoor environments. In the first phase, the coarse positions of the mobile devices were determined based on the maximum possible RSS. In the second phase, first, the angles between the PD and LEDs were estimated based on the ratio between the actual received power and the theoretical power that the receivers would receive if they are placed right under the LEDs (*i.e.*, when the incident angle of LED light is zero). This theoretical received power can be calculated from known parameters of LED and PDs. Then from the estimated AOA, the distances from the PD to the LEDs are calculated and finally quadratic programming was used to determine the fine position. The simulation results reported that an accuracy of 14 cm was achieved.

The work in [107] was extended in [108] by considering the mobility of receiver and the latency of position estimation. The paper applied a latency optimization to the system proposed in [107] to guarantee at least a position—coarse or fine, a position will be outputted as the result of the algorithm. More specifically, if the available information is insufficient (*i.e.*, when triangulation fails) and the latency exceeds a threshold, the algorithm yields the coarse estimate rather than a localization failure. Simulation showed that in any case, a coarse RSS based estimated position with 48 cm of accuracy was obtained rapidly within 17 nanoseconds and in most cases a fine AOA based estimated position with 14 cm of accuracy was achieved.

A proximity-RSS hybrid algorithm was proposed in [109]. In the first step, the coarse position of the mobile device was determined upon receiving the signal IDs of LED base stations. In the second step, a minimum mean square error (MMSE) algorithm was used to solve the RSS equations to find the refined position of the mobile device. Since the possible position of the mobile device was limited to the proximity area found in the first step, the MMSE algorithm only searched in much smaller area and thus consumed less power.

In practice, errors in positioning are likely to introduce disconnected estimated positions as the mobile device moves. In these cases, statistical methods such as a Kalman filter can be used to obtain smooth position tracking results. In the VLC based indoor positioning system proposed in [110], RSS was used to determine the position. And the tracking capability was enabled using particle and Kalman filters. The simulation results indicated that although it required longer time for processing, the particle filter yielded smoother results than the Kalman filter.

An indoor tracking system based on VLC and dead reckoning was proposed in [111]. At the beginning the position of the mobile device was determined based on VLC. After that, a dead reckoning process was performed using a Kalman filter and information from geomagnetic and gyro sensors attached to the mobile device. The experiment was conducted with a robot moving at 0.6 m/s and the reported angular and positioning errors were $6^{\circ }$ and 10.5 cm, respectively.

The authors of [112] introduced two methods for tracking the position of a robot. In the first method, a PD was used to receive the signal sent from LEDs at different frequencies. It was assumed that the robot has the prior knowledge about the location of all LEDs. Then by detecting the frequency of the received signal, the robot found its proximity. After that, a particle filter algorithm was applied to improve the positioning accuracy. In the second method, a camera and single view geometry algorithm were used to determine the position of the robot. The simulation results showed that the higher positioning accuracy was achieved by using camera.

In [113], a high speed camera and single view geometry algorithm were used for the initialization of a vehicle tracking system. Then an Extended Kalman filter was used for tracking the position of the vehicle. An experiment was conducted in an indoor environment with 8 LED placed in front of the vehicle. The result showed that the maximum distance error obtained in the initialization was less than 0.5 m.

In [114,115], a linear PD array installed horizontally on a vehicle was used for receiving the signal transmitted from LED traffic light. A convex-cylindrical lens was attached in front of the PD array to spatially separate different LED. An extrinsic parameter calibration was used to find the position of the vehicle. Since the linear photo array can sense the spatial information of the LEDs in one dimension, at least 4 LEDs must be in the view of the PD array to enable positioning. After this initialization step, an extended Kalman filter was used to track the position of the vehicle. Experimental result showed that the maximum distance error was less than 0.2 m.

### 3.6. Comparison of VLC Based Positioning Techniques

Basically, proximity has the simplest implementation, yet lowest accuracy among VLC based positioning algorithms. Fingerprinting can provide relative positions, which is more accurate than the proximity method but less accurate than the absolute position provided by triangulation and vision analysis algorithms. In general, the implementation of a fingerprinting algorithm is simple. However, preparing the fingerprinting database in the offline phase might be time consuming. The accuracy of a fingerprinting positioning system largely depends on how the fingerprinting database is prepared. In some cases, with an intricate database, fingerprinting can achieve an accuracy that is comparable to absolute algorithms.

TOA is considered to have the simplest mechanism among the absolute positioning algorithms. That is because after obtaining TOA information, the intersections of the TOA circles can be calculated easily to determine the position of the mobile device. Unfortunately, the TOA measurement is always subject to errors having three main causes. First, to measure the propagation time of the signal, the receiver must be perfectly synchronized with the transmitter. This is not easily done, even in an indoor environment. Second, the accuracy of the measured propagation time can be undermined due to the uncertainty of the sending time (*i.e.*, the time period for preparing the message) of the transmitter and the receiving time of receiver. Last but not least, the accuracy of the time measurement is limited to the resolution of the clock and the response of the PD. Because of these difficulties, a practical VLC based positioning system using TOA algorithm is expected to have low accuracy. This might be the explanation for the limited number of studies on VLC based positioning using TOA.

The mechanism of TDOA is more complicated than TOA since solving TDOA equations is known as a quite difficult problem. However, the implementation of a VLC based positioning system using TDOA would not introduce as much difficulties as the one using TOA. TDOA only requires the synchronization between transmitters, which is much more easily obtained than the synchronization between transmitters and receiver. It should be noticed that similar to TOA, TDOA also requires a very precise time measurement. In practice, even the limitation of the clock resolution and the PD response can cause errors in time measurements which severely degrade the positioning accuracy.

RSS, which is the most investigated algorithm for VLC based positioning system in the literature, has the advantage of asynchronous operation and simple implementation. Similar to TOA and TDOA, RSS positioning systems use a single PD as a receiver. This means that RSS has a low hardware cost. The most difficult part is creating a path loss model. Depending of the environment, the path loss models can be very different. Practically, the effect of irradiance angle and incidence angle are very difficult to be accounted for completely in the path loss model. Therefore, VLC based positioning systems using RSS usually work nicely when the receiver is horizontally near the LED, as the irradiance and incidence angle are small. When the receiver locates at a horizontally distant position to the LED, the irradiance and incidence are large, and thus the accuracy of RSS algorithm is degraded a lot. So in this regard, RSS is considered to not provide high precision.

Among the triangulation algorithms, AOA might yield the highest accuracy yet require the most complicated hardware and implementation. To obtain the angular information, multiple receivers and proper estimation mechanisms are usually required. In many cases, when the mobile device is rotatable, an additional inertial sensors such as gyroscope or 6-axis sensor is needed for the AOA measurement. Therefore, AOA is more expensive than the algorithms mentioned above. The bigger size of the receiver in AOA might be a limitation of AOA in implementation. Another disadvantage of AOA is that the estimation accuracy might degrade when the mobile device moves farther from the base stations. However, the precision of AOA is still considered to be higher than RSS.

Vision analysis is a promising algorithm for VLC based positioning. Compared to other algorithms, vision analysis utilizes the spatial separation ability of the image sensor and thus does not require any multiplexing scheme. Nowadays, any smart phone is equipped with a high resolution camera, meaning that they all ready to use vision analysis algorithms. The advantage of a vision analysis algorithm includes asynchronous operation, a simple mechanism and implementation, high accuracy and precision. The disadvantage of this algorithm is that a camera, which is much more expensive than a PD, must be used. In addition, the latency of a VLC based vision analysis positioning system might be longer than the requirement of some applications.

## 4. Types of Receivers and Multiplexing Techniques in VLC Based Positioning

### 4.1. Types of Receivers

Receivers have an important role in VLC based positioning systems. To determine the position, a plenty of useful information needs to be obtained. In most cases, the information about the location of the base stations must be known. If the base stations do not send the information, they could send their ID so that the mobile device can do a look-up in the database to obtain the information. The data rate greatly depends upon the type of receiver. Besides the IDs or locations of base stations, other information such as signal strength, signal strength ratio, time of arrival, or geometric information might be required for positioning algorithms. Each type of receiver has a different capability of obtaining the information. Therefore, depending on the positioning algorithm, different types of receiver will be used and vice versa—depending on the type of receiver, different positioning algorithm can be used.

#### 4.1.1. Photodiode

PDs have been widely used in VLC systems because of their low prices and the possibility of high reception bandwidths. PDs have also been adopted in most VLC based positioning systems, except those using vision analysis algorithms which require image sensors. In positioning, PDs have the advantages of the simplest hardware implementation and the ability to communicate with LED base stations at very high data rates. Generally, PDs can precisely measure the signal strength at high accuracy thanks to their high dynamic ranges. In addition, because PDs have fast response times, they can also measure the time of signal arrival at high accuracy. Therefore, PDs are suitable positioning systems using TOA, TDOA, or RSS algorithms.

On the down side, PDs can only offer the most basic information for positioning. Since PDs cannot separate light sources, multiplexing mechanisms are required when multiple LED base stations are used. PDs are also vulnerable to ambient light and thus are difficult to deploy successfully in outdoor environments.

#### 4.1.2. Image Sensors

Compare to PDs, image sensor can provide much more useful information related to the position of LEDs, to enable vision analysis algorithms. With the ability to separate light sources, image sensors inherently do not require any multiplexing mechanisms. Also, image sensors can stand against ambient light much better than a PD. However, image sensors have a big limitation related to data rate. To obtain the information, a sequence of grabbed images is processed to find the presence and absence of LEDs, which represent the logical 1 and 0, respectively.

Generally, the achieved data rate is less than or equal to the camera frame rate. Currently, the frame rate of a smart phone camera is 60 fps, which indicates a data rate limited to 30 bps when a single LED is used. Obviously, such a low data rate is insufficient for transmitting even the simplest positioning information. To achieve a higher data rate, a simple but expensive solution is to use a high speed camera to capture hundreds to thousands of image of LEDs per second. For communication purpose, the data rate should be as high as possible. To further increase the data rate, LED arrays can be used instead of LEDs to transmit light patterns which convey much more data in a single frame. For positioning purpose, the data rate doesn’t need to be very high and thus another solution, that is utilizing the rolling shutter effect of CMOS image sensors, can be used.

The technique of VLC using rolling shutter was proposed in [116] and analyzed in [117]. This technique is inexpensive in that it just requires a normal CMOS sensor camera which is equipped in most cameras and smart phones. However, to achieve a decent data rate, that is tens of kpbs, the distance from camera to LED must be very close. For example, the communication distance in [116,117] is less than 15 cm. Obviously, this distance is impractical for any positioning system. Fortunately, the required distance can be lengthened up to a few meters by using large LED panels or illuminated surfaces. The fingerprinting positioning system in [50] used white walls as the illuminated surfaces to achieve the working distance of a few meters. However, using large LED panels or illuminated surfaces cannot solve all the problems. For an absolute positioning algorithm using single view geometry, multiple LEDs must be in the view of the camera and thus the size of each LED in the image is small. Consequently, the achieved data rate can be very low. In [118], the camera on a smart phone used the rolling shutter effect to receive the signal transmitted from multiple LEDs. Experiments showed that a data rate of 1.25 bytes per second, which is just fast enough to send an ID code, was achieved. In some applications where the size of LEDs need to be small or the camera moves fast, this technique might not give a sufficient data rate for positioning.

#### 4.1.3. Photodiode Arrays

Besides PDs and image sensors, PD arrays can also be used to obtain more information compared to PDs and a higher data rate compared to an image sensor. The data rate offered by a PD array is equal or higher than that of a single PD. Although a PD array cannot provide sufficient information for a vision analysis algorithm, other algorithms such as fingerprinting and AOA which require more complicated signal strength information are possible with a PD array. In addition, timing information can be measured by a PD array as precisely as by a single PD. Therefore, a hybrid algorithm of fingerprinting, RSS, TOA, TDOA, and AOA can be possible using a PD array.

### 4.2. Multiplexing Technique

One might think that multiplexing has little to do with positioning. However, in most positioning systems, the position of the mobile device is determined based on the signals from multiple base stations. Therefore, a multiplexing technique must be applied in the positioning system. In fact, many studies on VLC based positioning only focus on proposing multiplexing techniques to use with existing positioning algorithms.

#### 4.2.1. Time Division Multiplexing

With time division multiplexing (TDM), different LED base stations would transmit their signals at different points of time. Compared to other multiplexing techniques, TDM has the advantage of simple implementation. However, it requires a longer time for receiving enough signals required for positioning, especially when there are many LED base stations. The biggest disadvantage of TDM is that all base stations need to be synchronized, which is not desirable in many positioning systems. To lessen the burden of synchronization, a random access mechanism like ALOHA can be used to avoid the synchronization problem. Another disadvantage of TDM is that it it is time consuming. With TDM, the number of LED base stations might be limited to prevent flickering.

#### 4.2.2. Frequency Division Multiplexing

Frequency division multiplexing (FDM) introduces complexity in designing the demultiplexer. However, with the advantage of asynchronous positioning, many systems used FDM. In these systems, the light in different LED base stations will be modulated to transmit at different frequencies. TDM and FDM used in VLC based positioning systems were compared in [119] and the authors came to the conclusion that the positioning accuracy provided by FDM is greater than that of TDM. It is worth noting that the simulation in [119] was conducted with the assumption that any two LEDs have a timing synchronization error of 10%.

In [120], the advantage of using OFDM, which is a specialized FDM, in an indoor VLC based positioning system was investigated. With OFDM, the visible light from different LEDs could be modulated at low symbol rates and thus the intersymbol interference, which might degrade the positioning accuracy, was mitigated. The simulation results showed that for the same system, using OFDM could achieve twice as much positioning accuracy compared to using OOK with TDM. Notice that in [120] the effect of intersymbol interference was clear since a high data rate up to 25 Mbps was assumed.

#### 4.2.3. Code Division Multiplexing

Code division multiplexing is more complicated compared to TDM and FDM. In [121], code division was shown to be more robust to noise sources compared to frequency division.

#### 4.2.4. Color Division Multiplexing

Color division multiplexing, which is also called wave length division multiplexing, uses LEDs consisting of a blue LED with a phosphor layer on top which makes it possible to change the light color. The problem with this method is that distinguishing different light colors is not as easy as generating different light colors due to the white balance of the image sensor. Furthermore, to make it easy for distinguishing colors, the number of colors used should be small. This would yield problems when many LED base stations are installed in a wide area.

#### 4.2.5. Space Division Multiplexing

Space division multiplexing can be used with any positioning system adopting an image sensor. For the multiplexing purpose, this approach is extremely effective. However, for positioning, the accuracy is affected by other factors such as sensor resolution and the optical quality of the lens. All VLC based vision analysis positioning systems apply this approach for multiplexing.

## 5. Summary of Studies in Visible Light Communication Based Positioning

Table 1 summarizes the positioning algorithms reviewed in this paper. Regarding the positioning accuracy, in some studies the achieved accuracies were not reported. In other studies, the reported accuracies were obtained through simulations or experiments. The reality of the assumptions in studies which tested their systems through simulations varied at various degrees. For studies which conducted real experiments, the testing environments were also very different. For example, some experiments were conducted in small boxes of less than 1 m${}^{3}$ while others were tested in large areas measuring hundreds of m${}^{2}$. Therefore, one should not compare the performance of algorithms listed in Table 1 based entirely on their reported accuracies. Similarly to accuracy, the used multiplexing techniques were not specified in some studies. However, there are also studies in which multiplexing is not needed. In such studies, a camera was used to separate signals or there was only one LED light source used for positioning.

## 6. General Difficulties in Visible Light Communication Based Positioning

### 6.1. Ambient Light Noise

Similar to VLC, VLC based positioning is also vulnerable to the ambient light noise. The luminance of a typical LED is hundreds times lower than that of sunlight. The authors in [122,123] showed that the PD became blind to LED when it was also exposed to direct sunligh. So far there is no effective solution for this problem. The author of [122] designed a simple light shield to conceal the PD from direct sunlight. In [123], an optical filter was used to block all light coming to the PD at un angle of $30^{\circ }$ or above. A more sophisticated approach, which canceled out the voltage caused by the sunlight was proposed in [124]. With all these approaches, the angle of view of the PD should be small and the PD needs to be directed toward the LED. These approaches are impractical for positioning purpose since the PD must receive the signal from multiple LEDs and thus cannot point toward a specific LED.

Compared to PD, image sensor does not suffer much from ambient light noise since the LEDs and the sun will be projected to different positions in the image. However, a strong light source can create haze and ghost in the image and thus detecting the position of the LED in the image would be more difficult. Besides sunlight, skylight is also an issue since the LED might not stand out from the sky background. Fortunately, LEDs might be installed on cars, bridges, or other dark backgrounds and this allows for the outdoor communication and positioning [125].

### 6.2. Time Measurement

Since the light travels at a very high speed, small errors in time measurement might lead to big errors in calculated distances. For example, an estimation error of 1 nanosecond might lead to a positioning error of 30 cm. In the indoor environment, the propagation time of light is only tens of nanoseconds and thus the accuracy requirement of the time measurement is even higher. In reality, it is very difficult to measure the time of arrival of a signal at such a high level of accuracy. Firstly the clock resolution must be very high, for example, at the sub-nanosecond level. Atomic clocks can provide such high accuracy, but their prices are unacceptable for most VLC based positioning systems. Secondly, even when the clock resolution is high enough, the measurement accuracy also depends on the rising and falling times of the LEDs, as well as the response times of the receivers. For example, the response time of a typical PD is a few nanoseconds. The method for measuring the time of arrival of the signal should take into account this amount of time to give an accurate result.

### 6.3. Out of Focus Images

When using the image sensor for positioning, the positioning of the LED in the image needs to be obtained with high accuracy. To achieve that, the image should be focused precisely [126]. When the LED is far from the image sensor, a small error in determining the LED positioning in the image can lead to a large error in the positioning accuracy. In some worst cases, different LEDs in the out of focus image can merge together and cannot be separated.

### 6.4. Synchronization

As mentioned before, synchronization between LED base stations and receivers is very difficult to obtain. Usually, wireless synchronization relies on some sort of data exchange to calculate the clock drift and clock offset. The accuracy of synchronization depends on the deterministic of the sending time, propagation time, and receiving time of the signal. However these quantities of time are usually obtainable with indefinite values. In [127], LEDs and the rolling shutter effect of the smart phone image sensor were utilized to achieve a synchronization accuracy of a few microseconds without exchanging data between transmitter and receiver. While the synchronization accuracy achieved by this method is quite impressive, it is still far from what would be necessary for obtaining decent positioning accuracy using visible light TOA.

The synchronization between LED base stations is much easier to obtain compared to the previous type of synchronization. To do that, however, wired connections between LED base stations are required, which might be undesirable for some applications.

### 6.5. Flickering

Similar to VLC systems, flickering should be avoided since the positioning systems also take over the function of illuminating. As mentioned before, flickering might occur when TDM is used with many LED base stations. Also, a cheap camera with a low frame rate might require the light to be modulated at low frequencies, which might introduce flickering. One of very few papers whose authors deal with the flickering problem in VLC based positioning is [128]. In general, flickering is not a very difficult issue but must be taken into account for any VLC positioning system.

### 6.6. Lens Distortion

**Figure 14:**
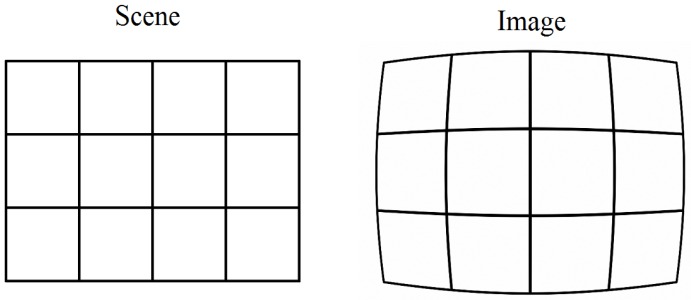
Lens distortion. (**Left**) Original scene; (**Right**) Distorted image.

One of the biggest issues of positioning using camera is lens distortion, which is illustrated in Figure 14. Because of the lens distortion, the LEDs will appear at wrong position on the image and thus the input of the positioning algorithm will contain errors regardless how good the image processing method is applied.

Any lens has some degree of distortion. Narrow angle lens usually has small distortion compared to wide angle lens. However for positioning purpose, the camera needs to be in the view of multiple LEDs and thus narrow angle lenses are inappropriate to be used. Some calibration process can be applied to solve the problem of distortion but it would lengthen the processing time of the positioning algorithm.

### 6.7. Trade-offs Regarding to FOV of Receiver and Image Sensor Resolution

Regarding the FOV of receiver, it is obvious that the positioning accuracy increases when the FOV decreases. However when the FOV is small, the receiver might not see enough LEDs for calculating the position and thus the robustness decreases when FOV decreases.

**Figure 15:**
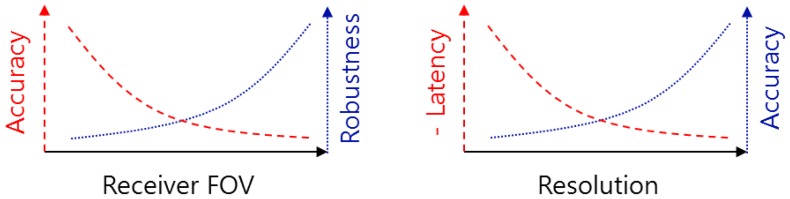
Trade-offs in VLC based positioning. (**Left**) Trade-off regarding to receiver FOV; (**Right**) Trade-off regarding to image sensor resolution.

Regarding the resolution of the image sensor, it is also obvious that the positioning accuracy increases when resolution increases. However when the resolution increases, beside the cost increases, the latency of the positioning process also increases, which is undesirable, especially when the mobile device is moving fast. (Figure 15) describes the two trade-offs mentioned above.

### 6.8. Mobility

The mobility issue in VLC based positioning was investigated in [129]. The simulation results showed that with a 3 W LED base station, an effective positioning radius of 1.3 m is achieved. Then given the bit rate set to 200 kbps, the paper showed that the maximum movement speed of a mobile device is 16.25 km/s. The paper also suggested that a higher bit rate should be applied to achieve steady positioning. In [73], not only the visible light ID but also the location information of LED base stations was transmitted to the receiver. Even though the system used PD, it took 2.1 seconds to receive all of the necessary signals for positioning. This latency might be unacceptable for applications where the subjects are not quite stationary.

## 7. Open Issues in VLC Based Positioning

Although precise positioning algorithms are the most investigated topic in the literature, proximity algorithms might be the one with the most potential for the thriving location based services and applications. The limitation of current VLC proximity systems is that they still require support from WiFi, ZigBee or other wireless networks to fulfill the positioning operation. For example, after receiving the visible light ID, the mobile device needs to send the ID to an external center to receive the position information. In the future, it would be desirable to have a VLC proximity system independent of RF wireless networks.

A topic, which is very attractive, yet difficult, is outdoor positioning based on VLC. Positioning using PDs still faces many problems in the outdoor environment. Using a camera, however, is very likely a possible solution for outdoor positioning and might be a hot research topic in the future. In addition, since every vehicle is equipped with high power front light and tail light, vehicle to vehicle positioning based on VLC and cameras is also very worth researching.

Since positioning systems only give disconnected positioning results, tracking systems might be more useful for moving objects. Most existing positioning systems in the literature do not have tracking ability. Therefore, adding tracking ability to current positioning systems is still an open issue.

In general, fundamental techniques such as modulation and multiplexing are ignored in many studies on VLC based positioning. However, these techniques are indispensable for any system and there are open issues of finding a new modulation technique to avoid flickering or to facilitate detection, or new multiplexing technique to shorten the positioning latency.

It would be appreciated if a system can undertake both positioning and communication function. However, such system might require clever designs of frame structures. For example, frame headers might be somehow utilized for positioning function. There is also the problem with modulation in such system. The high data rate required for communication would make the visible light signal suffer from the intersymbol interference issue, which might affect the positioning accuracy. Since most of the current positioning systems were designed for positioning purpose only, combining positioning and communication in a system is would be another open issue.

Nowadays, smart phones have shown rapid development with regard to processing power and camera function. Resolution is absolutely not a problem since the resolutions of current smart phone cameras are tens of mega-pixels, which are far more than enough to provide accurate positioning. Frame rates also seems not to be the problem when smart phones like iPhone 6 can provide a frame rate up to 240 fps. The processing power of a smart phone now is far more than enough to process any complicated positioning algorithm. Especially, smart phones are equipped with not only image sensors but also precise inertial sensors such as a gyroscope or 6-axis sensor, which can provide precious information for positioning. However, there are not many studies using smart phone for VLC based positioning and thus utilizing all of the powerful features of smart phones for VLC based positioning is another open issue.

Regarding positioning using cameras, there are many open issues that need to be researched more. For example, finding methods to calibrate the camera to deal with lens distortion, or to optimize the focal length of the lens and resolution of image sensor, considering the trade-offs between accuracy, robustness, and latency would be potential areas of research. Most of current image sensors including those on smart phones or consumer camera are CMOS sensors which adopt rolling shutter mechanism. Although the rolling shutter mechanism can be utilized for improving the data rate as mentioned before, it causes motion artifacts which might alter the position of LEDs in the image and thus degrades the positioning accuracy [130]. Existing VLC based positioning algorithms using camera all ignore this motion artifact. Therefore, finding mechanisms that mitigate this side effect of CMOS sensor would be another potential research area related to VLC based positioning using camera.

## 8. Conclusions

With many advantages such as energy efficiency, long lifetimes, ruggedness, environmental friendliness, and great controllability, LEDs have been considered to be the lighting device for the 21st century. Benefiting from the advantages of LEDs and the positive features of visible light, VLC using LEDs has also been considered to be the next generation of wireless communication technology that possibly enables the Internet of Things. The promising future of LEDs and VLC unfolds the potential of an omnipresent positioning system based on VLC. In the near future when VLC base stations are present everywhere, a positioning system based on VLC can provide a seamless positioning service with much larger coverage than current positioning technology. Besides the advantages that VLC based positioning inherits from LEDs and VLC, studies have shown that accurate positioning can be achieved with VLC based positioning. These prospects have caused a lot of researchers to propose new VLC based positioning systems. In this paper, an intensive survey of VLC based positioning literature was conducted. More than 100 papers ranging from pioneering papers to the state-of-the-art in the field were collected and classified based on different criteria. Current issues and trends regarding VLC based positioning were also discussed. 

## Figures and Tables

**Table 1:** Summary of different positioning algorithms.

Algorithm	Reference	Receiver	Multiplexing	Accuracy
Proximity	[8,21,22,23,24,25,26,27,28,30,33]	PD		
	[29]	PD		4.5 m
	[31,32]	PD		1–2 m
	[34,35,36]	PD + 6-axis sensor	no need	30–60 cm
	[37]	PD	FDM	12.9 cm
Fingerprinting	[39]	PD		0.81 cm
	[40]	PD	FDM	15–20 cm
	[41,42]	PD	TDM	20–80 cm
	[43,44]	PD	CDM	4.4–12.5 cm
	[45,46]	PD	TDM	1.58 cm
	[47,48]	PD array	no need	4–13 cm
	[49]	PD array	no need	6 cm
	[50]	PD + camera	no need	
TOA	[51]	PD	FDM	2–6 cm
RSS	[7]	PD	FDM	0.4 m
	[62]	PD	TDM	4–90 cm
	[63,64,65,66]	PD	FDM	6 cm
	[67,68]	PD + gyroscope		1.5 cm
	[69,70]	PD array	no need	1.5 cm
	[71]	PD	CDM	8 cm
	[72]	PD	FDM	1.5 cm
	[73]	PD	FDM	0.3–0.7 m
	[74]	PD	FDM	0.4 m
	[75]	PD		3–35 cm
	[76]	PD	TDM	3.2–10.3 cm
	[77]	PD	TDM	0.5–7.3 mm
	[78]	PD		5-10 cm
	[79]	PD	FDM	4.78 cm
	[80]	PD	CDM	6 cm
	[81]	PD		
	[82,83,84]	PD	TDM	1.7–3.9 cm
	[85,86]	PD	TDM	11–17 cm
	[120]	PD	FDM	0.53 m
	[121]	PD	CDM	0.7 m
TDOA	[52,53]	PD		14 cm
	[54,55]	PD	TDM	3 cm
	[56]	PD	TDM	68.2 cm
	[57]	PD	FDM	1 cm
	[58]	PD	FDM	1 cm
	[59]	PD	FDM	2 cm
	[60]	PD	FDM	
	[61]	PD	TDM	0.5–5 m
AOA	[87]	PD array		5–30 cm
	[88,89]	PD + accelerometer	TDM	25 cm
	[90]	PD array + accelerometer	TDM	6 cm
	[91]	PD + $10^{\circ }$ angle lens		tens of cm
	[92]	PD array	FDM	5 cm
	[93]	PD	OFDM	
Vision	[95]	camera	no need	
	[96]	camera	no need	
	[97]	camera	no need	7 cm
	[98]	camera	no need	10 cm
	[99]	camera + accelerometer	no need	5 cm
	[100]	camera	FDM	10 cm
	[101]	camera	no need	30 cm
	[102,103]	2 camera	no need	1.5 m
	[104]	2 camera	no need	85 cm
Hybrid	[105]	PD	TDM	5 mm
	[106]	PD array	no need	20 cm
	[107]	PD	TDM	14 cm
	[108]	PD	TDM	14–48 cm
	[109]	PD		
	[110]	PD		0.5 m
	[111]	PD + gyroscoper		10.5 cm
	[112]	camera	FDM	1 m
	[113]	camera		0.5 m
	[114,115]	camera		0.2 m
